# Convergent evolution of an extreme dietary specialisation, the olfactory system of worm-eating rodents

**DOI:** 10.1038/s41598-018-35827-0

**Published:** 2018-12-13

**Authors:** Quentin Martinez, Renaud Lebrun, Anang S. Achmadi, Jacob A. Esselstyn, Alistair R. Evans, Lawrence R. Heaney, Roberto Portela Miguez, Kevin C. Rowe, Pierre-Henri Fabre

**Affiliations:** 1 Institut des Sciences de l’Evolution (ISEM, UMR 5554 CNRS-IRD-UM), Université de Montpellier, Place E. Bataillon - CC 064 - 34095, Montpellier Cedex 5, France; 2 Museum Zoologicum Bogoriense, Research Center For Biology, Indonesian Institute of Sciences (LIPI), Jl.Raya Jakarta-Bogor Km.46, Cibinong, 16911 Indonesia; 3 Museum of Natural Science, 119 Foster Hall, Louisiana State University, Baton Rouge, Louisiana 70803 United States; 4 Department of Biological Sciences, Louisiana State University, Baton Rouge, Louisiana 70803 United States; 5 School of Biological Sciences, 18 Innovation Walk, Monash University, Victoria, 3800 Australia; 6 Sciences Department, Museums Victoria, Melbourne, Victoria 3001 Australia; 7 Field Museum of Natural History, 1400 S Lake Shore Drive, Chicago, 60605 United States; 8 Natural History Museum of London, Department of Life Sciences, Mammal Section, London, United Kingdom; 9 School of BioSciences, The University of Melbourne, Melbourne, Victoria 3010 Australia

## Abstract

Turbinal bones are key components of the mammalian rostrum that contribute to three critical functions: (1) homeothermy, (2) water conservation and (3) olfaction. With over 700 extant species, murine rodents (Murinae) are the most species-rich mammalian subfamily, with most of that diversity residing in the Indo-Australian Archipelago. Their evolutionary history includes several cases of putative, but untested ecomorphological convergence, especially with traits related to diet. Among the most spectacular rodent ecomorphs are the vermivores which independently evolved in several island systems. We used 3D CT-scans (N = 87) of murine turbinal bones to quantify olfactory capacities as well as heat or water conservation adaptations. We obtained similar results from an existing 2D complexity method and two new 3D methodologies that quantify bone complexity. Using comparative phylogenetic methods, we identified a significant convergent signal in the rostral morphology within the highly specialised vermivores. Vermivorous species have significantly larger and more complex olfactory turbinals than do carnivores and omnivores. Increased olfactory capacities may be a major adaptive feature facilitating rats’ capacity to prey on elusive earthworms. The narrow snout that characterises vermivores exhibits significantly reduced respiratory turbinals, which may reduce their heat and water conservation capacities.

## Introduction

Understanding how species have adapted to their environment is a major goal of evolutionary biology^1–3^. Salient examples of convergence, the evolution of a similar trait in independent evolutionary lineages^4^, have demonstrated the importance of determinism through natural selection^3^. Recent advances in X-ray microtomography (X-ray µCT) provide the opportunity to quantify convergence in morphological structures that are otherwise inaccessible^5–7^. In mammals, the use of morphological proxies such as inner ears, braincase, floccular fossa, cribriform plate, and turbinal bones^5–11^ have shed light on ecological and functional adaptations, especially for taxa that are difficult to observe directly in the wild^7^.

Extensive studies of the mammalian olfactory subgenome revealed that mammals have a wide array of olfactory receptor genes that represent 1–6% of their genomes^12–14^. The huge mammalian olfactory subgenome has proven useful to illustrate dietary and other adaptions^12,15,16^. However, the nasal chamber of mammals has been relatively neglected by anatomists due to its internal position^17^, and few studies have tested for an adaptive link between nasal morphology and olfactory capacities^18–20^.

**Figure 1:**
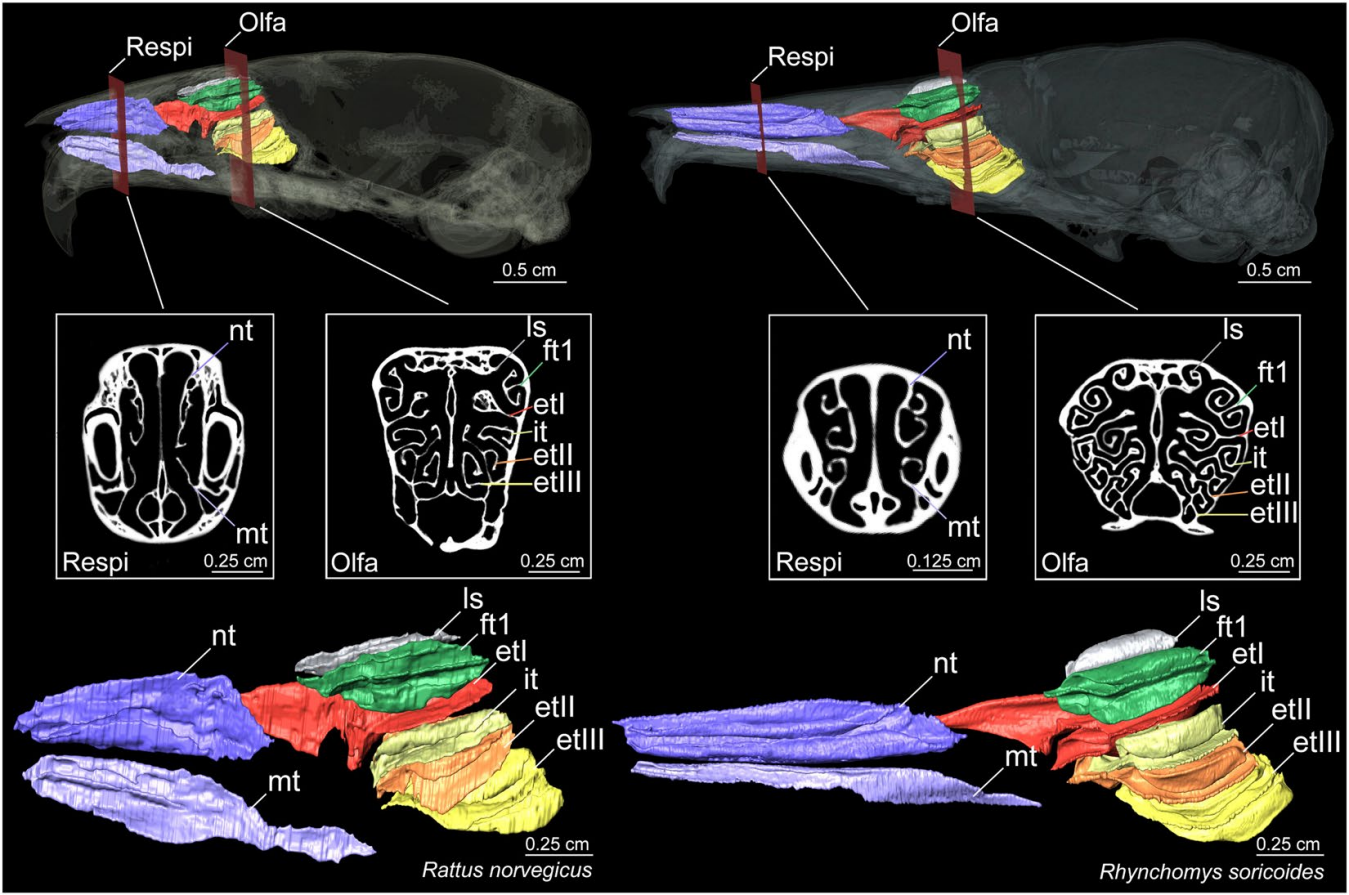
Coronal cross section and sagittal plane of skull and 3D representations of turbinal bones in *Rattus norvegicus* and *Rhynchomys soricoides*. Abbreviations: Respi = respiratory turbinals, Olfa = olfactory turbinals, nt = nasoturbinal, mt = maxilloturbinal, ls = lamina semicircularis, it = interturbinal, ft1 = frontoturbinal 1, etI = ethmoturbinal I, etII = ethmoturbinal II, and etIII = ethmoturbinal III.

In mammals, the nasal chambers contain bony plates called turbinals or turbinates, which are key structures involved in olfaction, thermoregulation, and water conservation. Because they bear the respiratory and olfactory epithelia^8,9,17,21–24^, these turbinals have played a major role in the evolution of homeothermy and olfaction in mammals^25^. Anatomists and physiologists usually distinguish two major functional parts for turbinals: (1) the respiratory and (2) the olfactory components (Fig. 1). The respiratory turbinals, which are anterior to the olfactory turbinals, are further divided into maxilloturbinals (MT) and nasoturbinals (NT, Fig. 1). Maxilloturbinals link the naris and nasopharynx and are covered by respiratory epithelium, a vascularised mucosa. During inhalation, they moisten and warm the breath; at exhalation, they conserve moisture^17,23,26–29^. Nasoturbinals are located in the anterior portion of the nasal cavity, near the naris, and dorsal to the MT. They contribute to homeothermy, as suggested by their distal position from the olfactory bulbs, the presence of respiratory epithelium, airflow dynamics, and performance tests^21,22,27,29–33^. However, NT probably also serve, at least partly, as olfactory structures^34^ because they are partially covered by olfactory epithelium in groups such as rodents^24^. As such, NT probably serve dual functions for olfaction and heat and water conservation^21,22^. The olfactory turbinals are primarily associated with the olfactory process. They are covered by a thick olfactory epithelium, innervated by several olfactory receptors and directly connected to the close cerebral olfactory bulbs via olfactory nerves^9,17,23,24,35–37^. In addition, olfactory turbinals are divided into several structures variably named lamina semicircularis (ls), frontoturbinals (ft), interturbinals (it), and ethmoturbinals (et, Fig. 1)^38–41^.

Studies of carnivorans suggest a possible link between olfactory turbinal size and olfactory performance as well as between respiratory turbinal size and heat or moisture conservation performance^8,9,42^. The physiological importance of turbinal bones was thereby shown by the correlation between surface areas of these bones and species’ ecological traits. These studies have especially demonstrated the correlation between dietary adaptations and the surface area of olfactory turbinals^23^. However, these types of studies are rare outside of carnivorans, leaving open the question of whether connections between ecological traits and turbinal surface areas is a general pattern.

Rodents of the family Muridae have migrated from mainland Asia to the many islands of the Indo-Australian Archipelago (IAA) multiple times since the Miocene^43,44^. These small to medium-sized mammals have spread over most of the IAA, where they occupy many terrestrial niches^43,44^. Included among this diversity are the “shrew-rats”, carnivorous rodents (i.e., those that feed on metazoans) that evolved independently in New Guinea, the Philippines, and Sulawesi^44–47^. Shrew-rats are an ideal comparative system to study dietary specialisation because they have convergently evolved from an ancestral omnivorous diet toward carnivory^44^. This adaptation appeared at least five times in the highly diverse Murinae, with at least two origins of highly specialised carnivorous lineages: (1) the Sulawesi shrew-rats and (2) the Philippine shrew-rats. Several species of shrew-rats consume a wide-range of invertebrates, but others are earthworm specialists with spectacular changes to their rostrum morphology. In the most specialised vermivorous species (*Paucidentomys* and *Rhynchomys* genera), the snout is extremely long and narrow^44,45,48^ and might have constrained the size and shape of turbinals. Additionally, the snout morphology of these vermivores might involve increased olfactory capacities to detect earthworms in leaf-litter.

Using comparative phylogenetic methods, we contrasted turbinal surface area and turbinal complexity between vermivorous, carnivorous and omnivorous species of Murinae to test hypothesised adaptations related to olfaction and heat and moisture conservation in the shrew-rats. We tested for convergence of the vermivorous pattern. In doing so, we propose two new indices of three dimensional (3D) complexity of turbinal bones, which we have implemented in the freeware MorphoDig^49^.

## Results

### Turbinal surface area

**Figure 2:**
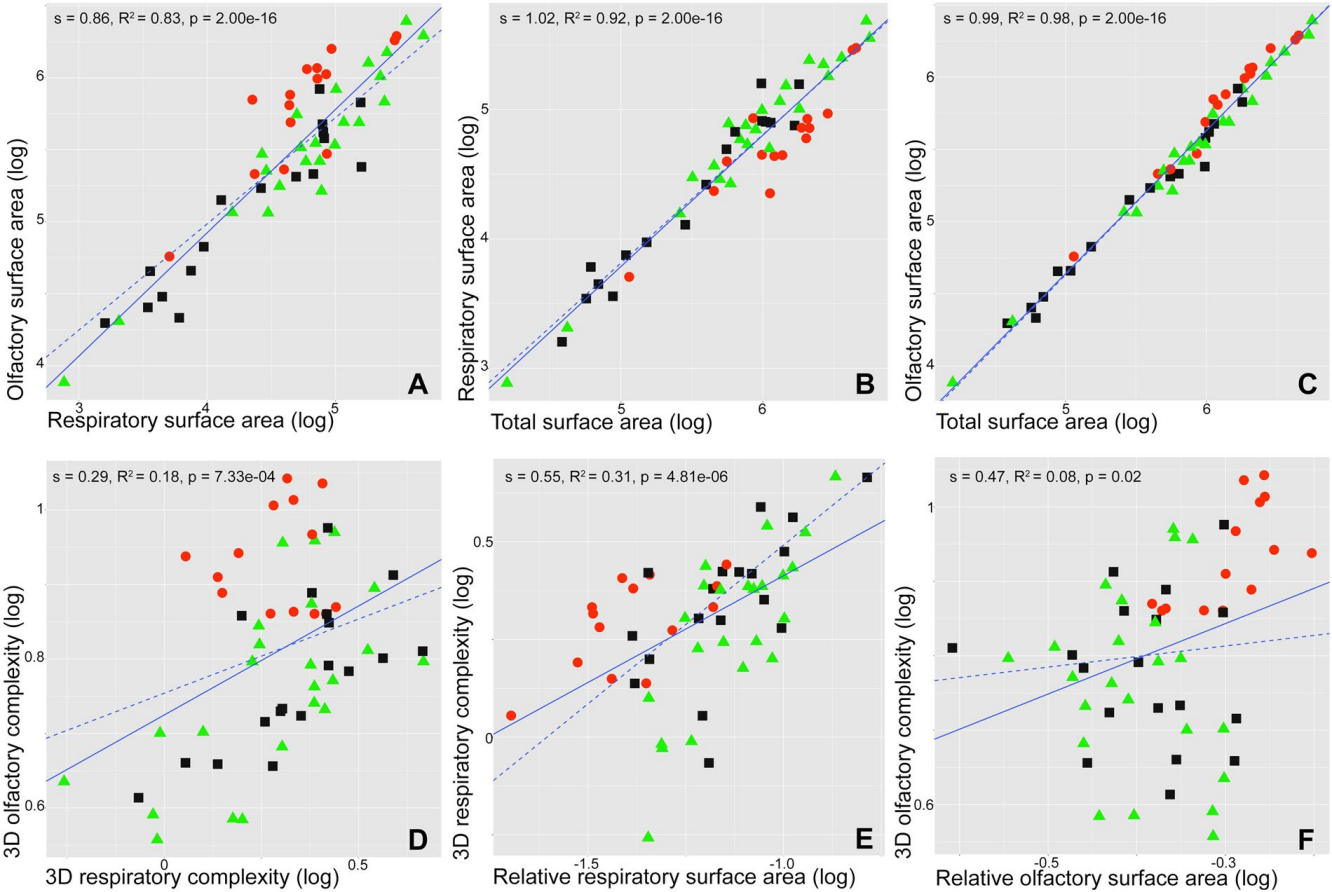
Log–log regressions (continuous line) and PGLS (dashed line) of (**A**) olfactory vs respiratory turbinal surface area, (**B**) respiratory vs total surface area, (**C**) olfactory vs total surface area, (**D**) olfactory vs respiratory 3D complexity (CHAR), (**E**) respiratory 3D complexity (CHAR) vs relative respiratory surface area, and (**F**) olfactory 3D complexity (CHAR) vs relative olfactory surface area. Colours and symbols: red dots = vermivorous, black squares = carnivorous, and green triangles = omnivorous.

There is a significant correlation between surface area of all turbinals and skull length (electronic supplementary material (ESM), Fig. S3A; slope (s) = 2.25, r squared (R^2^) = 0.88, p-value (p) = 2.00e-16). The surface area of all turbinals show strong positive allometry (s = 2.25). The PGLS slope of vermivores is significantly different from the PGLS slope of carnivores and omnivores (p = 0.01 and 0.02, respectively; ESM, Fig. S3A). This indicates that when skull length increases, vermivores have a smaller increase in turbinal surface area than do carnivores and omnivores. This surface area difference could be explained by the smaller area of the respiratory turbinals (ESM, Fig. S3B). In fact, the PGLS slope of respiratory turbinal area and skull length in vermivores is significantly different from that of carnivores and omnivores (p = 0.01 in both cases; ESM, Fig. S3B). Furthermore, there are no PGLS slope differences between dietary categories for the correlation between olfactory turbinal surface area and skull length (p > 0.05; ESM, Fig. S3C). There is a significant correlation between olfactory and respiratory surface area (Fig. 2A; slope (s) = 0.86, R^2^ = 0.83, p = 2.00e-16) and these variables display a negative allometry (s = 0.86). There are significant correlations between the surface area of respiratory or olfactory turbinals and the surface area of all turbinals (Fig. 2B and C; s = 1.02, R^2^ = 0.92, p = 2.00e-16 and s = 0.99, R^2^ = 0.98, p = 2.00e-16, respectively). PGLS slopes do not differ significantly between dietary categories for these two correlations (p > 0.3; Fig. 2B, C) and the relationship between these variables is isometric (s = 1.02 and s = 0.99; Fig. 2B, C). This suggests that sampled species exhibit the same relationship for these variables, thereby allowing comparisons of respiratory and olfactory turbinals between dietary categories.

**Figure 3:**
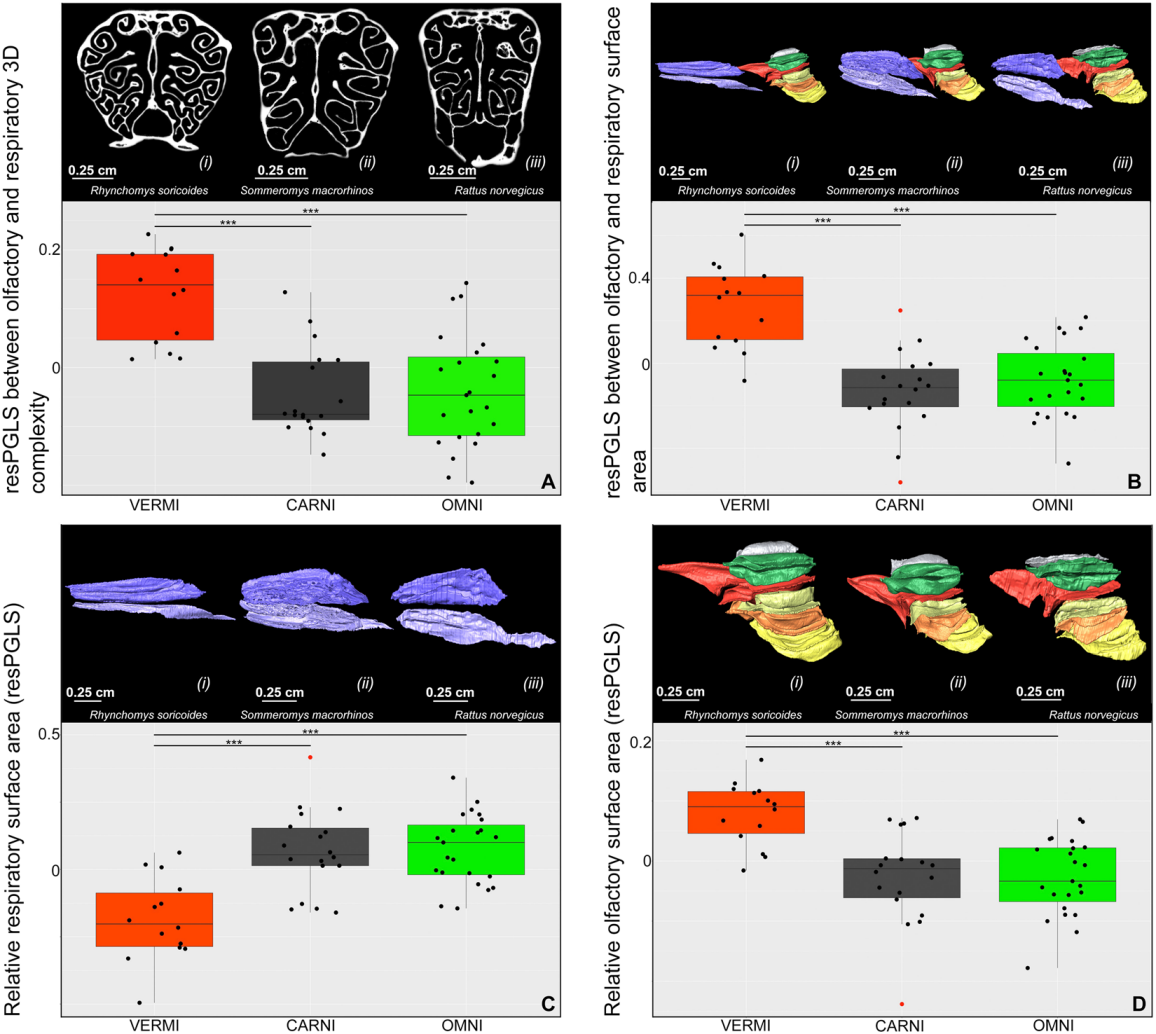
Boxplot with dietary categories: (**A**) residuals of PGLS (resPGLS) between olfactory and respiratory 3D complexity of turbinals (CHAR), (**B**) resPGLS between olfactory and respiratory surface area, (**C**) relative respiratory surface area, and (**D**) relative olfactory surface area. Significance codes are based on phylogenetic Tukey’s HSD test. (i) *Rhynchomys soricoides*, (ii) *Sommeromys macrorhinos*, and (iii) *Rattus norvegicus*. Colours: red = vermivorous, black = carnivorous, and green = omnivorous. Red points are outliers.

ANOVA reveals that the residuals of PGLS (resPGLS) between olfactory and respiratory turbinals surface area is significantly affected by diet (p = 2.62e-07; ESM, Table S2). Indeed, vermivores have resPGLS between the surface area of olfactory and respiratory turbinals significantly higher than carnivores and omnivores (Fig. 3B; p = 1.00e-04; ESM, Table S3). Phylogenetic ANCOVA shows similar results with significant differences between vermivorous and carnivorous dietary categories (p = 1.43e-08; ESM, Table S6). Considering the nasoturbinal either as olfactory or as respiratory turbinals does not significantly change the results (ESM, Table S4). Moreover, slope differences between linear regressions and PGLS are small (ESM, Table S1). Differences between phylogenetic and non-phylogenetic Tukey’s HSD tests are also small (ESM, Table S5).

ANOVA reveals that the relative surface area of olfactory turbinals is significantly affected by diet (p = 2.62e-07; ESM, Table S2). Indeed, vermivores have significantly higher relative surface area of olfactory turbinals as compared to carnivores and omnivores (Fig. 3D; p = 9.17e-05 and 8.40e-05, respectively; ESM, Table S3). Phylogenetic ANCOVA shows similar results with significant differences between vermivorous and carnivorous dietary categories (p = 8.46e-07; ESM, Table S6).

ANOVA reveals that the relative surface area of respiratory turbinals is significantly affected by diet (p = 4.11e-06; ESM, Table S2). Indeed, vermivores have significantly smaller relative respiratory turbinal surface area, relative nasoturbinal surface area, and relative maxilloturbinal surface area than do carnivores and omnivores (Fig. 3C; p = 1.18e-05, 1.00e-05, 2.67e-04, 2.19e-04, 4.47e-03, and 1.34e-03, respectively; ESM, Fig. S4A, B, Table S3). Phylogenetic ANCOVA shows similar results with significant differences between vermivorous and carnivorous dietary categories (p = 8.46e-07; ESM, Table S6).

### Turbinal complexity

Olfactory and respiratory 3D complexity are significantly correlated (CHAR, Fig. 2 D; s = 0.29, R^2^ = 0.18, p = 7.33e-04). Olfactory 3D complexity (CHAR) and skull length are also significantly correlated (ESM, Fig. S3E; s = 0.27, R^2^ = 0.27, p = 2.60e-05). ANOVA reveals that resPGLS between olfactory and respiratory 3D complexity (CHAR) is significantly affected by diet (p = 3.59e-07; ESM, Table S2). Indeed, vermivores have a significantly higher resPGLS between the 3D complexity (CHAR) of olfactory and respiratory turbinals compared to carnivores and omnivores (Fig. 3A; p = 2.70e-06 and 1.60e-06, respectively). Phylogenetic ANCOVA shows similar results with significant differences between vermivorous and carnivorous dietary categories (p = 1.82e-03, ESM, Table S6). Slope differences between linear regressions and PGLS are low (ESM, Table S1). 3D complexity (CHAR) of olfactory turbinals is significantly affected by diet (p = 8.54e-05). Indeed, vermivores and carnivores express a significantly higher olfactory turbinal complexity than omnivores (p = 5.42e-05 and p = 0.05, respectively; ESM, Table S3). Respiratory turbinals 3D complexity (CHAR) is not significantly affected by diet (p = 0.14; ESM, Table S2).

Results obtained with our two 3D complexity indices are similar to each other (ESM, Fig. S5 and Table S3) and to those obtained from 2D complexity (ESM, Fig. S5 and Table S3). This indicates that the 2D complexity signal from the middle slice of each turbinal group extracts the complexity of each turbinal group.

### Turbinal surface area and turbinal complexity

**Figure 4:**
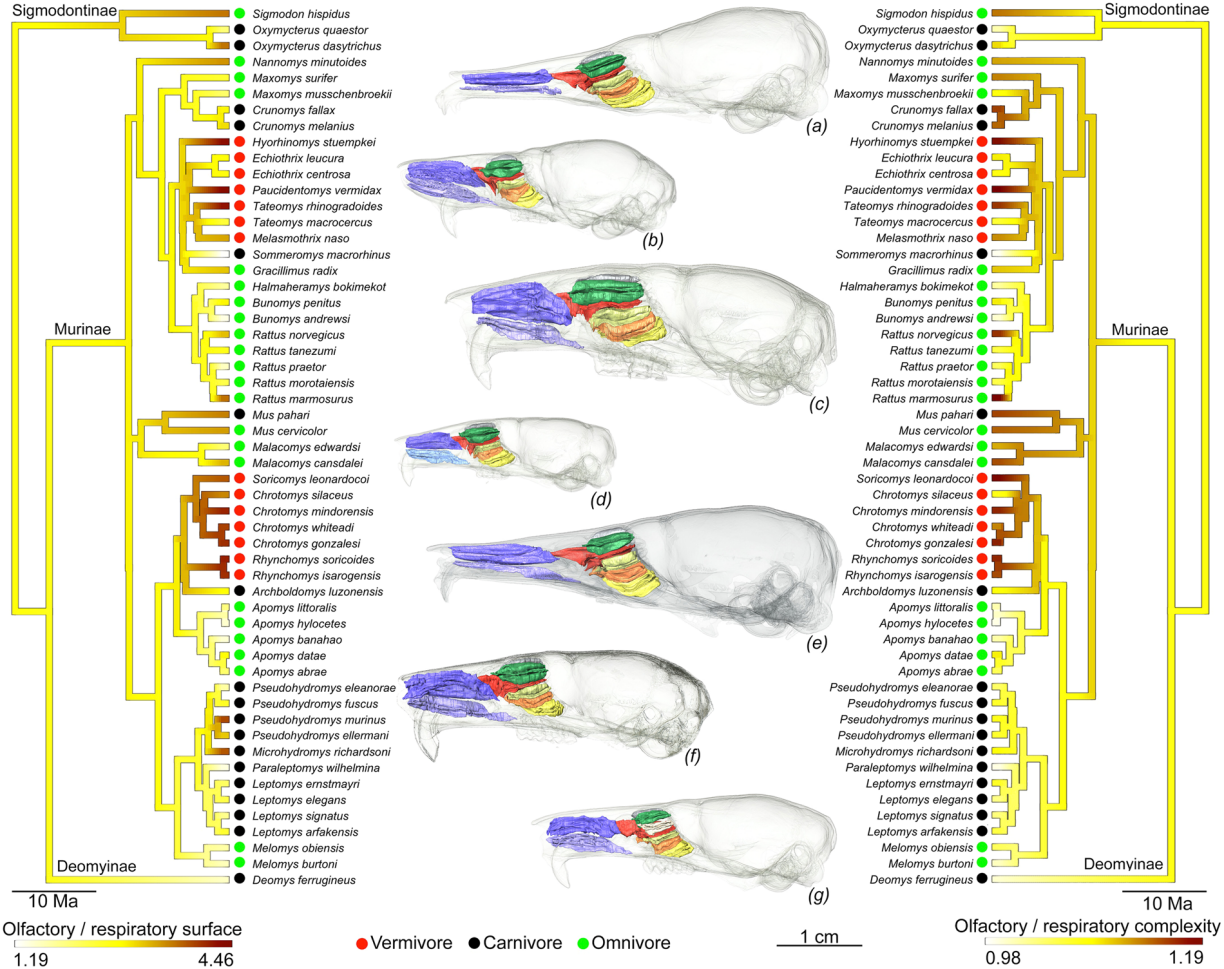
Continuous mapping of the ratio between olfactory and respiratory turbinal surface area (left) and of the ratio between olfactory and respiratory turbinal 3D complexity (CHAR, right) with phylogenetic relationships. (**a**) *Paucidentomys vermidax*, (**b**) *Sommeromys macrorhinos*, (**c**) *Bunomys penitus*, (**d**) *Mus pahari*, (**e**) *Rhynchomys soricoides*, (**f**) *Apomys banahao*, and (**g**) *Deomys ferrugineus*.

There is a significant correlation between 3D complexity (CHAR) and relative surface area of respiratory turbinals (Fig. 2E; s = 0.55, R^2^ = 0.31, p = 4.81e-06). Considering phylogeny, there is no significant correlation between the 3D complexity (CHAR) and the relative surface area of olfactory turbinals (Fig. 2F; PGLS s = 0.14, PGLS p = 0.36). The continuous phylogenetic mapping of the ratio between olfactory and respiratory surface areas and 3D complexity (CHAR) reveals similar patterns for both proxies (surface area and complexity; Fig. 4). However, four species display a different pattern between these two proxies: *Chrotomys silaceus*, *Maxomys surifer*, *Microhydromys richardsoni*, and *Pseudohydromys murinus* (Fig. 4). Even if patterns between surface area and 3D complexity (CHAR) are similar (Fig. 4), very low R^2^ values in some PGLS (Fig. 2; ESM, Fig. S3, and Table S1) reveal that we need to consider both proxies to understand olfactory capacities.

### Snout

Snout length and width differences are significantly affected by diet when vermivores are separated into two ecological subcategories: terrestrial and semi-fossorial vermivores (p = 0.01, p = 1.09e-04, respectively; ESM, Table S2). Indeed, semi-fossorial vermivores (*Chrotomys* spp.) have significantly shorter snouts than carnivores and terrestrial vermivores (p = 0.04, p = 0.03, respectively; ESM, Table S3). Terrestrial vermivores have significantly narrower snouts than omnivores and semi-fossorial vermivores (ESM, Fig. S6; p = 0.02 and 6.03e-05, respectively; ESM, Table S3). Semi-fossorial vermivores (*Chrotomys* spp.) have significantly larger relative snouts compared to omnivores, carnivores, and terrestrial vermivores (ESM, Fig. S6; p = 0.01, 2.00e-03, and 6.03e-05, respectively; ESM, Table S3).

### Adaptation and convergence

The best-fitting model is OU2 (Table 1), a model with 3 adaptive optima: omnivorous, carnivorous, and vermivorous dietary categories for (A) relative respiratory surface area, (B) relative olfactory surface area, (C) olfactory and respiratory surface area, (D) 3D complexity (CHAR) of olfactory turbinals, and (E) 3D complexity (CHAR) of olfactory and respiratory turbinals. The best fitting model for the relative snout width is OU3 (ESM, Table S7), with 4 adaptive optima: omnivorous, carnivorous, terrestrial vermivorous, and semi-fossorial vermivorous diets and lifestyles.

**Table 1:** Results of 1 000 simulations of single-rate BM and three alternative OU models with (A) the ratio between respiratory and total surface area, (B) the ratio between olfactory and total surface area, (C) the ratio between olfactory and respiratory surface area, (D) the 3D olfactory complexity (CHAR), and (E) the ratio between olfactory and respiratory 3D complexity (CHAR).

Model	(A) RespiSA/TotSA		(B) OlfaSA/TotSA		(C) OlfaSA/RespiSA		(D) OlfaCHAR		(E) OlfaCHAR/RespiCHAR	
	AICc	ΔAICc	AICc	ΔAICc	AICc	ΔAICc	AICc	ΔAICc	AICc	ΔAICc
BM	−132.63	34.35	−132.63	34.35	453.76	1127.27	−94.73	15.01	−127.42	62.83
OU1	−161.79	5.20	−161.79	5.29	−668.09	5.42	−107.18	2.56	−186.47	3.78
OU2	−166.99	0.00	−166.98	0.00	−673.51	0.00	−109.74	0.00	−190.25	0.00
OU3	−164.58	2.40	−164.59	2.40	−669.04	4.47	−107.50	2.25	−185.76	4.49

BM and OU1 with omnivorous and all carnivorous dietary categories (carnivorous + vermivorous); OU2 with omnivorous, carnivorous, and vermivorous dietary categories; and OU3 with omnivorous, carnivorous, terrestrial vermivorous, and semi-fossorial vermivorous dietary categories. AICc = Akaike’s information criterion corrected. ΔAICc = difference between AICc compared to minimum AICc.

Considering the C2 index, vermivorous murine convergence is highly significant when we test for global rostral pattern composed of relative olfactory and respiratory surface area, olfactory 3D complexity (CHAR), and snout width (Table 2). The convergence is not significant when we consider the C1 and C3 indices (Table 2). Considering the three indices (C1, C2, and C3), the convergence is highly significant when we test for the relative olfactory surface area, the respiratory surface area, and the olfactory 3D complexity (Table 2); or when we test for the relative olfactory surface area and the olfactory 3D complexity (Table 2).

**Table 2:** Results of the three convergence index tests as proposed by Stayton 2015^106^ with: RelatOlfaSA = relative olfactory surface area, RelatRespiSA = relative respiratory surface area, OlfaCHAR = olfactory 3D complexity of the convex hull area ratio, and SNW = snout width.

Variables	C1	p-value	C2	p-value	C3	p-value
RelatOlfaSA + RelatRespiSA + OlfaCHAR + SNW	0.233	0.123	0.11	0.002	0.122	0.113
RelatOlfaSA + RelatRespiSA + OlfaCHAR	0.394	0.003	0.163	<1.000e-04	0.207	0.005
RelatOlfaSA + OlfaCHAR	0.408	0.009	0.165	<1.000e-04	0.217	0.003

## Discussion

### Olfactory capacities in vermivorous murines

Compared to carnivores and omnivores, vermivores should have significantly better olfactory capacities, based on both the larger surface area and higher complexity of their olfactory turbinals. We hypothesised that these bony specialisations are related to an improvement of their olfactory adaptations allowing them to detect prey that are underground or invisible within wet leaf litter (Heaney, pers. comm). Such prey may be especially elusive and difficult to detect for more generalist, opportunistic rats. Indeed, molecular odorants are especially difficult to detect underground as compared to on the surface^50^. Most of these insular vermivorous rats (*Melasmothrix*, *Soricomys*, and *Tateomys*) are terrestrial and display relatively long claws in order to dig into moss, bark, leaf litter, and damp soil, where these earthworms are most abundant^51^. Other earthworm specialists patrol runways (*Echiothrix* and *Rhynchomys*)^45,52^ or dig underground (*Chrotomys*)^52^ to find their prey. The wide and short snout of the semi-fossorial vermivorous *Chrotomys* (ESM, Table S3 and Fig. S6) might be a fossorial adaptation that is also found in other fossorial rodents like chisel-tooth diggers^53,54^. Following Heth & Todrank^50^, the semi-fossorial vermivorous *Chrotomys* should have higher olfactory capacities than terrestrial ones, in order to detect molecular odorants from their underground prey. Based on turbinal complexity and surface area measurements some vermivores display the most derived morphology relative to the sampled murines with the highest olfactory capacities. This occurs with semi-fossorial species (*Chrotomys* spp.), with species that are patrolling along runways (*Rhynchomys isarogensis* and *R. soricoides*), that dig into bark (*Tateomys rhinogradoides*) and some with unknown feeding behaviours (*Hyorhinomys stuempkei* and *Paucidentomys vermidax*; Figs. 3 and 4). As our results show, the morphological diversity of these vermivores is quite large and we have a rather limited knowledge about their ecological diversity. Ecological studies of rodents are difficult due to most species’ nocturnal activity, poor trapping success, and sometimes low abundance, especially in the case of the vermivores^55–57^. It will be important in the future to investigate in greater detail stomach contents using metabarcoding if we are to understand the link between olfactory capacities and dietary behaviors of vermivorous rats. Different ecomorphs of earthworm specialists might occur in different underground and ground layers. Our results reveal a connection between dietary specialisations and surface area and complexity of olfactory turbinals, which suggests a functional link. However, these lines of morphological evidence are just a first step toward understanding the ecological and functional diversity of shrew-rats. While the link between the size of olfactory organs or the number of olfactory receptors and olfactory performance is debated^9,18–20,58,59^, mammals show a strong correlation between the size of a morphological proxy for olfaction (the cribriform plate) and the repertoire of olfactory receptor genes (OR)^11^.

To our knowledge, there is no study showing a clear relation between olfactory performances and the size of olfactory proxies such as turbinal bones, cribriform plate, olfactory bulb or vomeronasal organ. This lack of knowledge does not allow us to discriminate between acuity, sensitivity, and discrimination when we used olfactory turbinal proxies. However, our findings about the highly specialised vermivores suggest that an increasing in olfactory turbinal size is probably not correlated with odorant acuity, that is the ability to detect a wide array odorants^9^. Integrative studies of the olfactory system that include performance tests will further our understanding of these distinctive animals.

### Heat and moisture conservation

In terrestrial vermivores, the distal part of the snout is narrow (ESM, Table S3 and Fig. S6), which is assumed to be a morphological adaptation to earthworm consumption^60–62^. Such snout morphology has profound consequences for the respiratory surface and complexity of turbinals. Under a trade-off hypothesis between olfactory and respiratory turbinals, respiratory turbinal reduction could be a consequence of the increased size of olfactory turbinals. Indeed, previous work on carnivorans^9^ suggests a trade-off between olfactory and respiratory turbinal areas due to the limited rostral space and the need for other functions, such as vision or cranio-mandibular muscles. Additionally, the highly specialised cranio-mandibular apparatus of vermivores^48^ might impact the evolution of their rostrum, and the narrowing trend has resulted in highly reduced surface of the naso- and maxilloturbinal bones (Fig. 3C and ESM, Table S3 and Fig. S4). Depending on the organism and their environmental conditions, the respiratory turbinals may be involved in water conservation (e.g., in salty or dry environments) or heat retention (e.g., in cool or aquatic environments)^8,17,26–32,63^. Despite wide altitudinal and thermal differences in the sampled murines, the reduction of heat and moisture conservation potential in vermivores may not present a major energetic constraint in their tropical and terrestrial environments. Under the trade-off hypothesis between respiratory and olfactory turbinals, respiratory turbinal reduction might have facilitated an increase of olfactory capacities as the novel cranio-mandibular specialisations developed in vermivorous lineages.

### Vermivores convergence

Claims of convergence were previously proposed for vermivorous murines based on discrete character observations^44,51^, or by the use of a common vernacular name: shrew-rats. Dietary convergences in both insular and continental murids were recently demonstrated with stomach content evidence^44^. Using a large-scale phylogenetical framework for murids, Rowe^44^ inferred ancestral dietary state and recorded at least 7 shifts from an omnivorous to a carnivorous diet, with a potential reversal from carnivory to omnivory in *Gracilimus*^64^. Our results demonstrate a strong convergence footprint involving aspects of both the rostrum and turbinal morphologies (Tables 1, 2, and ESM, Table S7). Specifically, convergence among shrew-rats involves larger and more complex olfactory turbinals (Fig. 3A, B, D, 4, and ESM, Table S3), reduced respiratory turbinals (Fig. 3C and ESM, Table S3 and Fig. S4), and narrower snouts (ESM, Table S3 and Fig. S6). As explained in previous sections, these convergent patterns are probably related to dietary adaptations within the most specialised vermivorous forms.

Convergence among these shrew-rats might have been fostered by their replicated colonisation of islands in the Indo-Australian Archipelago, a hypothesis that is in accordance with the insular adaptive radiation theory^65,66^. However, colonisation of islands is not the only factor that might have led to the convergence of these lineages that are mainly found on the largest islands with mountainous landscapes^52,64^. Indeed, most IAA vermivores occur at relatively high elevation^47,52,67–69^. This distribution pattern coincides with an increase in earthworm density and abundance, demonstrated along elevation transects both in Luzon (Philippines) and Borneo (Malaysia)^55,70^. Rowe *et al*.^44^ suggested that the altitudinal distribution of vermivores might be explained by increased earthworm abundance as well as the reduction of potential food competitors such as ants that are most abundant in the lowlands^55,71,72^. Richness and abundance of small mammals is also higher at high altitude in islands of the IAA^57,72–75^. Inter-specific interaction of small mammals is another hypothesis to explain the diversity of these vermivores, especially on islands. Both high species richness and high competition for resources in these small mammal communities might have fostered these convergences. In fact, their omnivorous ancestors independently took advantage of an ecological niche that was likely vacant, mainly in an insular context. Specialisation into shrew-rat ecomorphs (runner, digger, and fossorial) might have reduced food competition and allowed co-occurrence of several earthworm specialists that likely share diverse earthworm resources at mid- to high elevations on islands. The successful dietary specialisation of vermivores was associated with independent acquisitions of large and complex olfactory turbinal bones that presumably improved olfactory capacities. Beyond the morphological convergence of molar reduction^62^ and turbinal bones, other convergent aspects will certainly be revealed by future anatomical and functional studies.

## Conclusion

Despite recent studies about mammal olfaction^11,16,76,77^ our knowledge in this field is rather limited. For example, the olfactory and respiratory epithelial covers are unknown or poorly described in most of non-model species. Comparative histology will help to refine the functional discrimination between olfactory and respiratory turbinals. Additionally, very few studies have been done concerning the complexity of turbinal bones^8,9,28,77,78^. Consequently, further studies will be necessary to understand the functional role of the complexity in nasal airflow and odorant deposition^79^. Despite this first evidence showing the possible trade-off between respiratory and olfactory turbinal bones (Fig. S3) further studies should use other variables than the skull length to test this hypothesis. Indeed, skull length might covary with nasal cavity and turbinal bones. Finally, other anatomical proxies should be further investigated such as the nasal septum, the cribriform plate, the olfactory bulb or the vomeronasal organ to understand multiple factors of murine olfaction.

Turbinal bones are important structures to understand how species that are challenging to study in the field have adapted to their environment. Consequently, museum specimens with undamaged turbinates are very valuable. Over the past few years there is an emerging trend to request samples of turbinal bones from museum specimens for molecular work (R. Portela Miguez in pers.). In light of the findings of our research, we recommend that the integrity of these nasal structures should be preserved so others can replicate this study or investigate other species applying similar methods.

## Material and Methods

We borrowed 87 skulls belonging to 55 rodent species from: American Museum of Natural History (AMNH), Centre de Biologie et de Gestion des Populations (CBGP), Field Museum of Natural History (FMNH), Museums Victoria (NMV), Museum Zoologicum Bogoriense (MZB), Natural History Museum London (NHMUK), Natural History Museum of Paris (MNHN), Smithsonian Institution National Museum of Natural History (NMNH), and University of Montpellier (UM). These samples comprised 14 vermivorous (30 specimens), 18 carnivorous (28 specimens), and 23 omnivorous species (29 specimens (ESM, Tables S8 and S9)). All sampled species were considered terrestrial except for *Chrotomys*, a semi-fossorial genus^52,55,56^. For outgroups, we selected additional carnivorous and omnivorous genera in Cricetidae (*Oxymycterus* and *Sigmodon*) and Muridae (Deomyinae).

### Digitising and measurement

Skulls were scanned using X-ray microtomography on a SkyScan 1076 (ISEM Institute, Montpellier), Nikon Metrology HMX ST 225 (NHMUK Natural History Museum, London), or SkyScan 1174v2 (The Evans Evolutionary Morphology Lab, Monash University, Melbourne). Acquired voxel size ranged from 18 to 36 μm. We digitised each left turbinal from each individual with Avizo Lite 9.0.1 software (VSG Inc., Burlington, MA, USA). This process was completed by semi-automatically selecting and delimiting each turbinal on each reconstructed virtual slice. Segmentation followed turbinal descriptions presented for Rodentia^80^, Lagomorpha^38^, and Marsupialia^81^. According to these references, we divided into 8 or 9 turbinals (Fig. 1 and ESM, Figs. S1 and S2) and followed anatomical terminology of ontogeny^38–41^. For the lamina semicircularis, we segmented only the homologous branching part (Fig. 1 and ESM, Figs. S1 and S2) that is covered by olfactory epithelium^24^. We identified an additional frontoturbinal (ft2) positioned between ft1 and etI (ESM, Fig. S2), which is only present in the outgroups (Deomyinae and Sigmodontinae). A second interturbinal (it) was also found in one individual of *Tateomys macrocercus* (Murinae). These additional turbinals were used in quantitative analyses of olfactory surfaces because they are located in the olfactory recess and should be covered by epithelial olfactory cells as are other olfactory turbinals^24^. Following previous comparative studies works that used turbinal bone surface area^9,23^, we decide to not include other bone structures that are covered by epithelium other than turbinals. For example, the nasal septum is partially covered in sensory epithelium^17,24,82^ but accurate delimitation is not possible with dry skulls. For all following quantitative measures and analyses, we took species averages for which we have multiple specimens (ESM, Table S8).

Skull length (SKL) was measured between the most anterior part of the nasal bone and the most posterior part of the occipital bone^83^. Snout length (SNL) was measured between the most anterior part of the nasal bone and the posterior-most portion of the naso-frontal suture. The snout width (SNW) was measured across the nasolacrimal capsules^83^. Length measurements were exported using Avizo Lite 9.0.1 software (VSG Inc., Burlington, MA, USA).

### Turbinal surface area

We divided the turbinals into olfactory and respiratory regions to estimate the surface area available for these two functions and used the surface area as a proxy for olfactory or heat and moisture conservation capacities. Due to the impossibility of estimating the proportion of nasoturbinal that was involved in olfaction or in heat and moisture conservation, we performed separate surface area analyses including nasoturbinal either as respiratory or as olfactory turbinals (ESM, Table S4). Within turbinal regions, we assumed that the different epithelial cells and receptors were evenly distributed and as such, greater surface area indicates greater capacity. We sized turbinal surface areas by the total surface area of all turbinals. The surface area of segmented turbinals were exported using Avizo Lite 9.0.1 software (VSG Inc., Burlington, MA, USA).

### Turbinal complexity

In addition to surface area, we also used turbinal complexity as a proxy for olfactory and heat or moisture conservation capacities. We interpret complexity as the degree of details in a predefined area. Following the principles of fluid dynamics, proportionally more fluid volume will come in contact with the edge of a narrow pipe than in a larger pipe. We assume that the same rule applies to air as it passes by the turbinals. As such, turbinals should be more efficient for surface exchange in complex structures than in simpler ones. As an example, a species with a high olfactory turbinal complexity is hypothesised to have good olfactory capacities.

To measure 2D turbinal complexity, we used the box counting method^84,85^. The complexity value (Db) was based on the number of boxes placed into a grid and necessary to cover the shape border, changing box size from large to small. It is a ratio between the details and the total scale, quantifying the fractal dimension of the bone. To simplify the process of 2D complexity acquisition, we measured turbinal complexity for each respiratory and olfactory turbinal group. We considered that all anteriorly positioned turbinals (respiratory turbinals) were involved in heat and moisture conservation, while posterior ones (olfactory turbinals) participated in olfaction. Using ImageJ software^86^, we extracted scanned images corresponding to the middle of the total number of slices composing each turbinal group. We converted the turbinal shape into a single pixel-wide binary contour using skeletonisation. The image was then scaled and centered onto a 300 × 300-pixel black square with Adobe Photoshop CS6 software. Images were converted to grayscale and binary formats. The 2D complexity value was obtained with ImageJ plugin FracLac^87^. Slice surface area was used as a size proxy to scale complexity values.

To measure 3D turbinal complexity we propose two indices implemented in the freeware MorphoDig^49^. These indices both make use of 3D convex hulls. A convex hull is the smallest convex envelope that contains the studied shape, in our case the turbinal bones.

Firstly, the convex hull area ratio (CHAR) is the ratio between the turbinal surface area (SA) and the surface area of the corresponding convex hull (CHSA):

$$CHAR=\frac{SA}{CHSA}$$

Secondly, the convex hull normalised shape index (CHNSI) measures how much turbinal surface area (SA) can be enclosed within the volume defined by the convex hull of the turbinal (CHV). It is defined as:

$$CHNSI=F\frac{\sqrt{SA}}{\sqrt[3]{CHV}}$$

where F is a constant defined so that spherical shapes express a CHNSI index equal to 1, as the 3D convex hull of a given:

$$F=\frac{\sqrt[3]{\frac{4}{3}\pi }}{2\sqrt{\pi }}$$

### Quantitative analyses

We performed phylogenetic generalized least squares (PGLS) using R v.3.2.4^88^, with ape^89^, nlme^90^, and phytools^91^. The phylogeny used for the following analyses was adapted from Fabre *et al*., Rowe *et al*., and Steppan & Schenk (^43,44,92^, ESM, legend S1). To determine if slopes were significantly different between dietary groups and to compare allometric effects, we performed analysis of covariance (ANCOVA) following Claude^93^. We also performed analysis of variance (ANOVA) based on the residuals of PGLS (resPGLS) to test for dietary influence on turbinal surface area, turbinal complexity, snout length, and snout width. To test for group differences, we performed the multiple comparison test of Tukey’s HSD based on the residuals of the PGLS with the R package multcomp^94^. To compare differences without phylogeny we also performed Tukey’s HSD tests based on the residuals of linear regressions. For all analyses based on PGLS we performed model fitting with: a model without phylogeny, Brownian (BM), Ornstein–Uhlenbeck (OU), and Grafen models in order to adapt the phylogenetic model to our data^95,96^.

Because methodological studies pointed out some biased results when residuals are treat as data^97–99^, we compared our residual approach with phylogenetic ANCOVA (ESM, Table S6). We contrasted three models: a model without dietary categories (H0), a model with omnivorous and carnivorous dietary categories (Carni), and a model with omnivorous, carnivorous, and vermivorous dietary categories (Vermi). Models were compared using the Akaike information criterion (AIC) and the Likelihood-ratio test (LRT).

### Adaptation and convergence tests

To test for associations between dietary categories and turbinal surface area, turbinal complexity, snout length, and snout width, we fit Brownian motion (BM) and Ornstein–Uhlenbeck (OU) models^96,100,101^. We computed 1,000 simulations of single-rate BM and three alternative OU models: BM and OU1 with omnivorous and all carnivorous dietary categories (carnivorous + vermivorous); OU2 with omnivorous, carnivorous, and vermivorous dietary categories; and OU3 with omnivorous, carnivorous, terrestrial vermivorous, and semi-fossorial vermivorous dietary categories. Model fits were compared using differences in the Akaike information criterion (ΔAIC). If earthworm consumption had a deterministic impact on the evolution of one of our measured traits, the best-fitted models should be OU2 or OU3. We ran these analyses with R packages: ape^89^, corpcor^102^, mvMORPH^100^, phytools^91^, and subplex^103^.

To visualize a pattern of convergence in surface area and complexity states, we separately mapped the ratio between olfactory and respiratory turbinal surface area and complexity on the phylogeny. Using maximum likelihood (ML)^104^, we estimated ancestral states at internal nodes and interpolated the states along each edge of the phylogeny^105^.

To quantify convergence among dietary groups in turbinal surface area, turbinal complexity, snout length, and snout width, we used three measures proposed by Stayton^106^. Firstly, C1 is the inverse of the ratio between the phenotypic distance between convergent tips (Dtip) and the maximum distance between any pair of taxa in those two lineages (Dmax). Secondly, C2 is the difference between Dmax and Dtip. Thirdly, C3 is the ratio between C2 and the sum of all phenotypic distances from ancestors to descendants (Ltot.clade). Contrary to C2 and C3, C1 compares phenotypic similarities and phylogenetic relationships without taking into account the absolute amount of evolution that has occurred during convergence^106^. We ran these analyses with a modified R package convevol^107,108^, performing 1,000 simulations.

## Electronic supplementary material

Supplementary information

## Data Availability

Raw data are available in the electronic supplementary material (ESM). The CT-scan surfaces could be requested to the corresponding author.
