# Supplementary material for: Convergent evolution of an extreme dietary specialisation, the olfactory system of worm-eating rodents
Source: Sci Rep. 2018 Dec 13;8:17806. doi: 10.1038/s41598-018-35827-0 (PMC6293001; doi:10.1038/s41598-018-35827-0)
Supplement: Supplementary file 1 — Supplementary information [file 41598_2018_35827_MOESM1_ESM.pdf]

Electronic supplementary material (ESM) for:

Convergent evolution of an extreme dietary specialisation, the olfactory system of worm-eating rodents

Quentin Martinez[1*], Renaud Lebrun[1], Anang S. Achmadi[2], Jacob A. Esselstyn[3,4], Alistair R. Evans[5,6], Lawrence R. Heaney[7], Roberto Portela Miguez[8], Kevin C. Rowe[6,9], and Pierre-Henri Fabre[1]

[1] Institut des Sciences de l'Evolution (ISEM, UMR 5554 CNRS-IRD-UM), Université de Montpellier, Place E. Bataillon - CC 064 - 34095 Montpellier Cedex 5, France

[2] Museum Zoologicum Bogoriense, Research Center For Biology, Indonesian Institute of Sciences (LIPI), Jl.Raya Jakarta-Bogor Km.46 Cibinong 16911, Indonesia

[3] Museum of Natural Science, 119 Foster Hall, Louisiana State University, Baton Rouge, Louisiana 70803, United States

[4] Department of Biological Sciences, Louisiana State University, Baton Rouge, Louisiana 70803, United States

[5] School of Biological Sciences, 18 Innovation Walk, Monash University, Victoria 3800, Australia

[6] Sciences Department, Museums Victoria, Melbourne, Victoria, 3001, Australia

[7] Field Museum of Natural History, 1400 S Lake Shore Drive, Chicago, 60605, United States

[8] Natural History Museum, London, Department of Life Sciences, Mammal Section, United Kingdom, London

[9] School of BioSciences, The University of Melbourne, Melbourne, Victoria, 3010, Australia

*Corresponding author (quentinmartinezphoto@gmail.com).

Figure S1. Coronal cross section and sagittal plane of skull and 3D representations of turbinal bones in *Rattus norvegicus*, Rhynchomys soricoides, and *Paucidentomys vermidax*. Abbreviations: see Table S1.

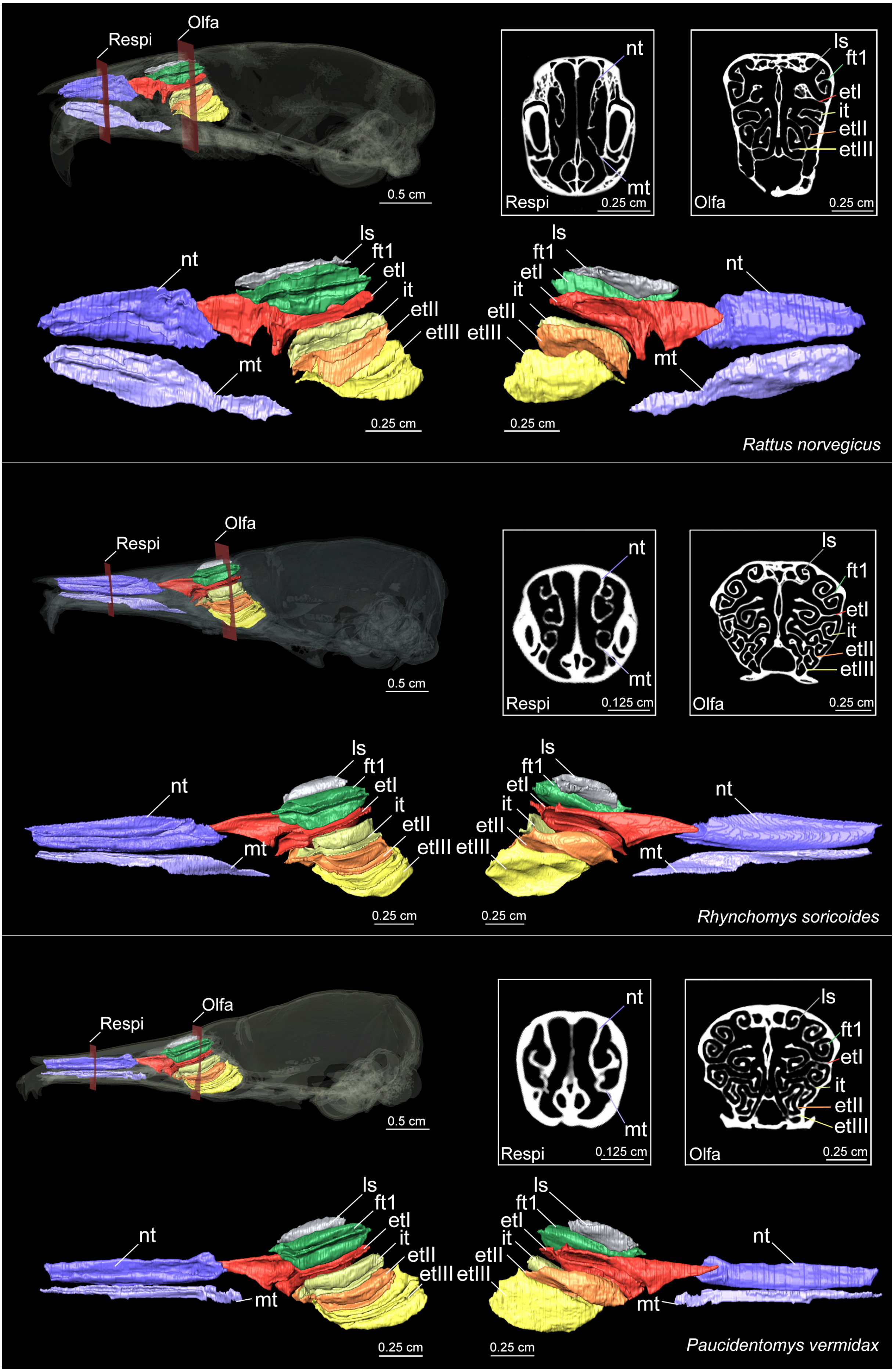

Figure S2. Coronal cross section and sagittal plane of skull and 3D representations of turbinal bones in *Sommeromys macrorhinos*, *Deomys ferrugineus*, and *Mus cervicolor*. Abbreviations: see Table S1.

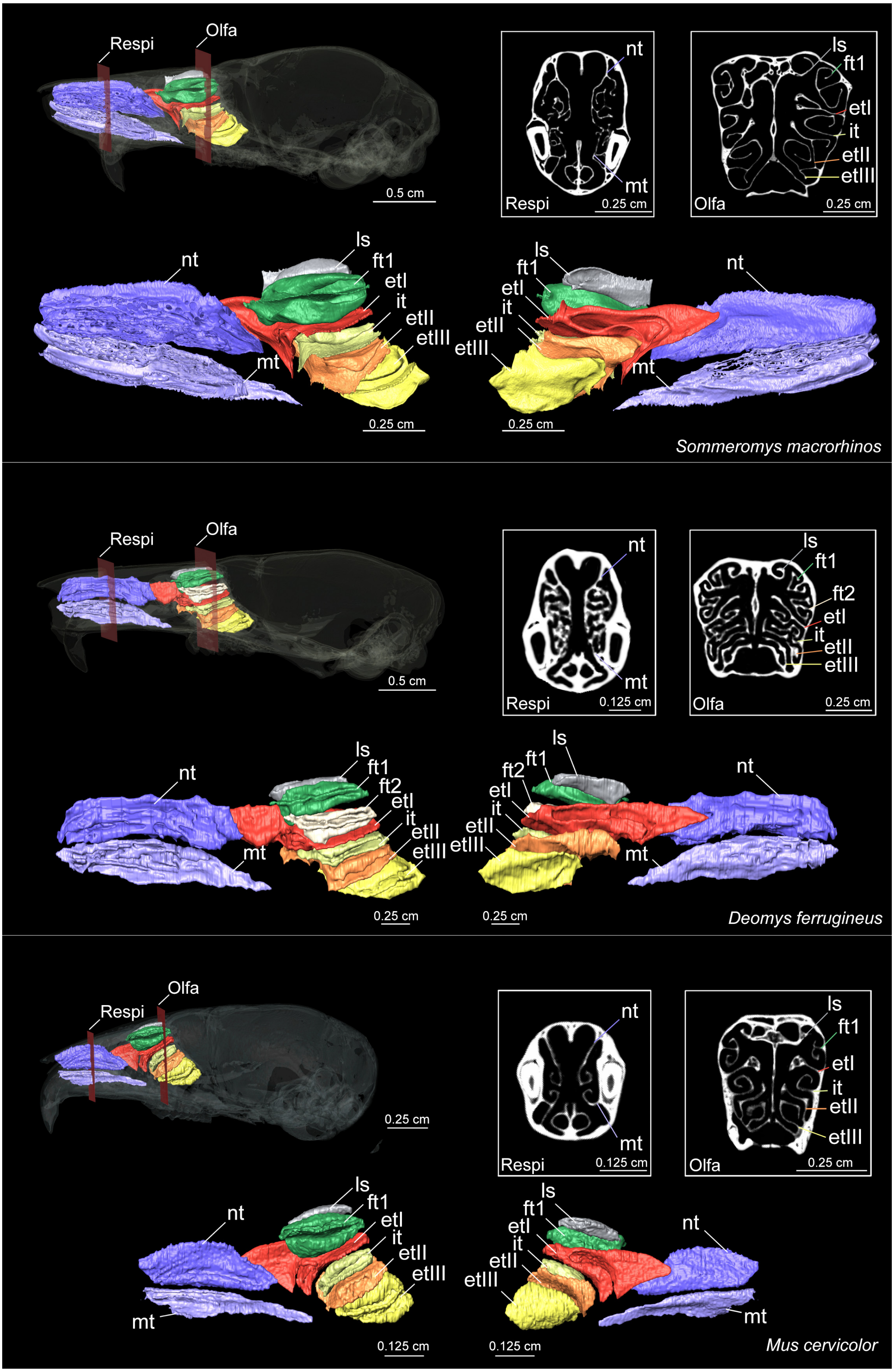

Figure S3. Linear regressions (continuous line) and PGLS (dashed line) of (A) total surface area of turbinals against skull length, (B) respiratory turbinals surface area against skull length, (C) olfactory turbinals surface area against skull length, (D) 3D respiratory complexity (CHAR) against skull length, and (E) 3D olfactory complexity (CHAR) against skull.

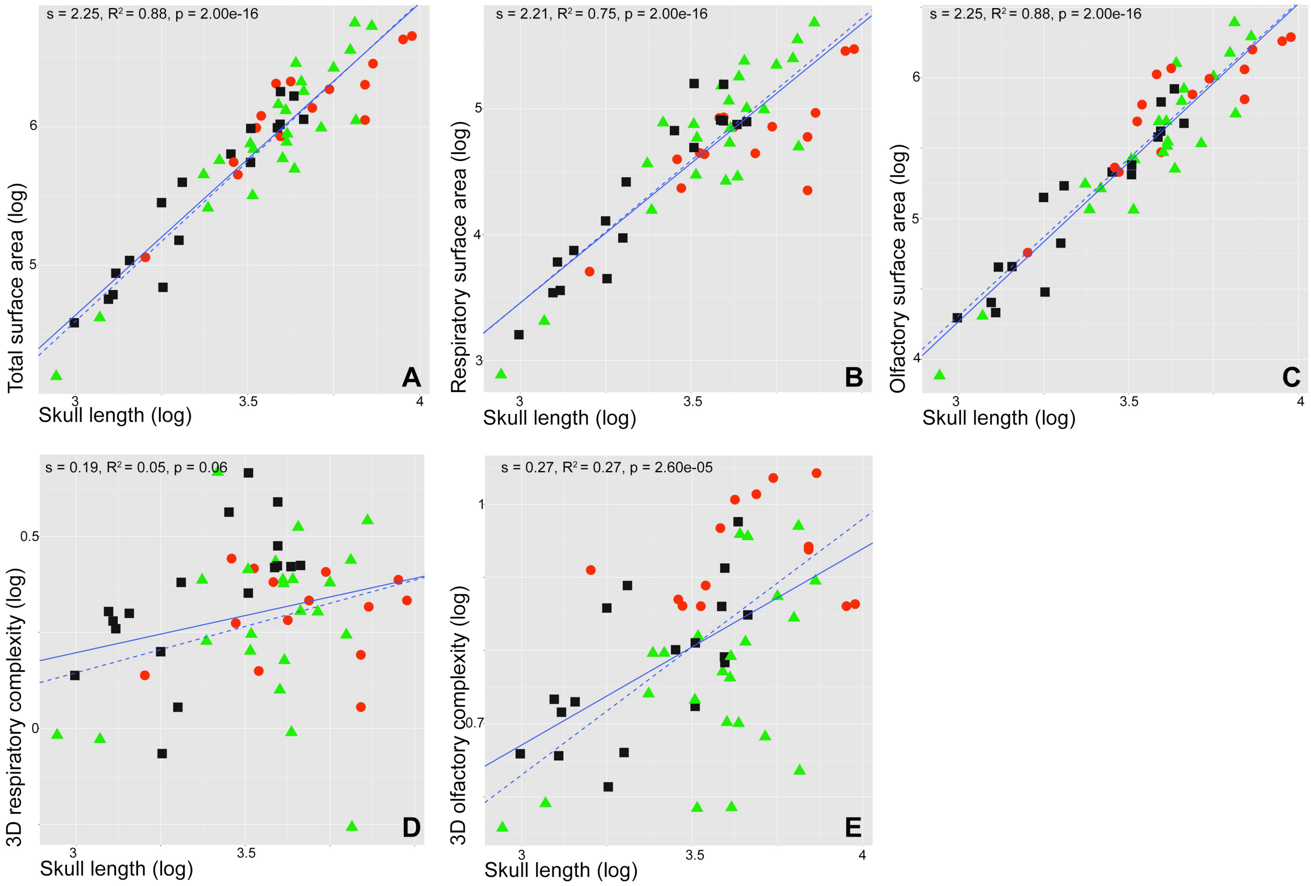

Figure S4. Boxplot with dietary categories: (A) relative nasoturbinal surface area and (B) relative maxilloturbinal surface area. Boxplot are based on PGLS residuals. Significance code are based on phylogenectic Tukey's HSD test. (i) *Rhynchomys soricoides*, (ii) *Sommeromys* macrorhinos, and (iii) *Rattus norvegicus*. Red points are outliers.

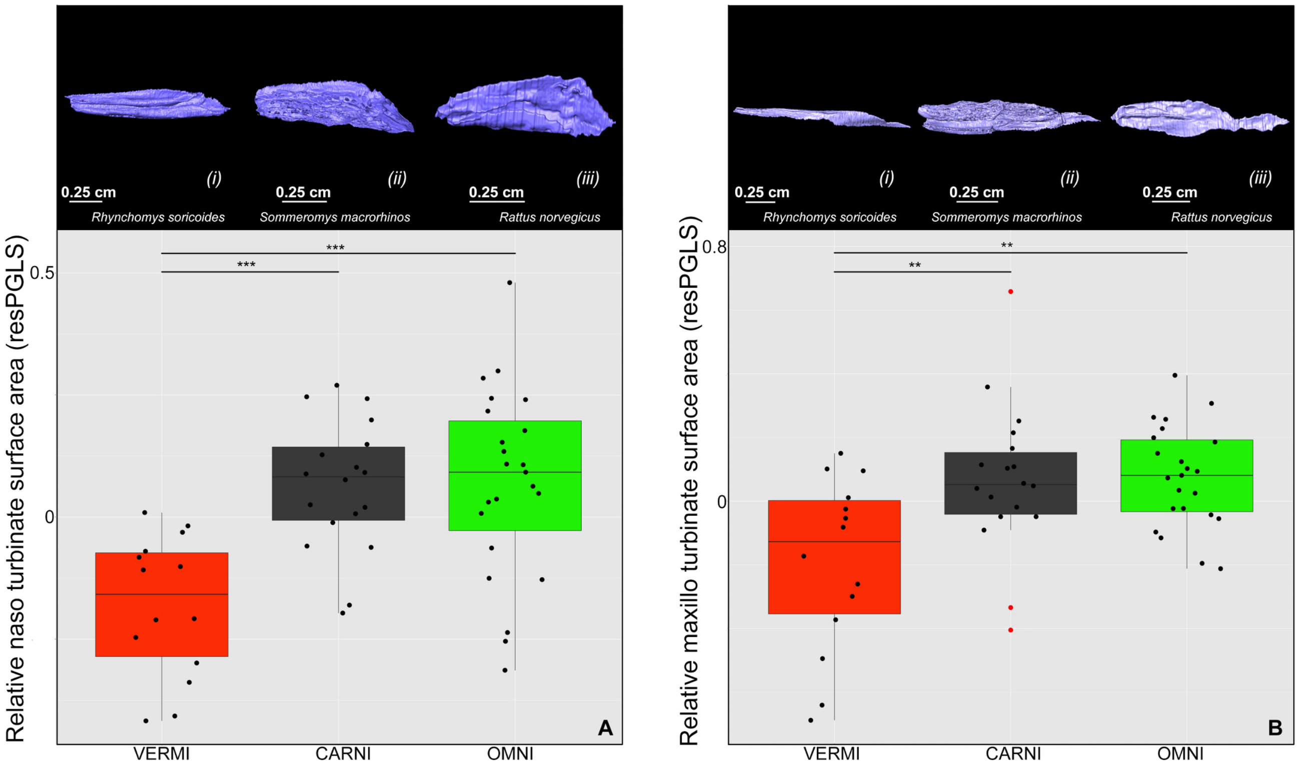

Figure S5. Boxplot of the residuals of PGLS (resPGLS) between olfactory and respiratory complexity with dietary categories: (A) 2D complexity method, (B) 3D complexity method (CHAR), and 3D complexity method (CHNSI).

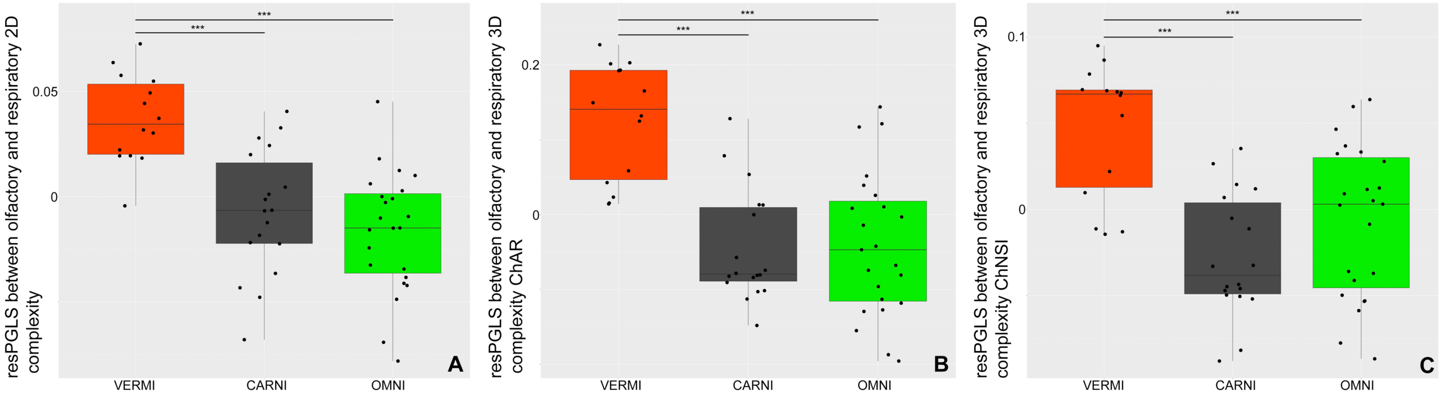

Figure S6. Boxplot of snout width (SNW) with dietary categories. Boxplot are based on PGLS residuals. Significance code are based on phylogenectic Tukey's HSD test. (i) *Rhynchomys isarogensis,* (ii) *Leptomys elegans,* (iii) *Malacomys cansdalei,* and (iiii) *Chrotomys silaceus.*

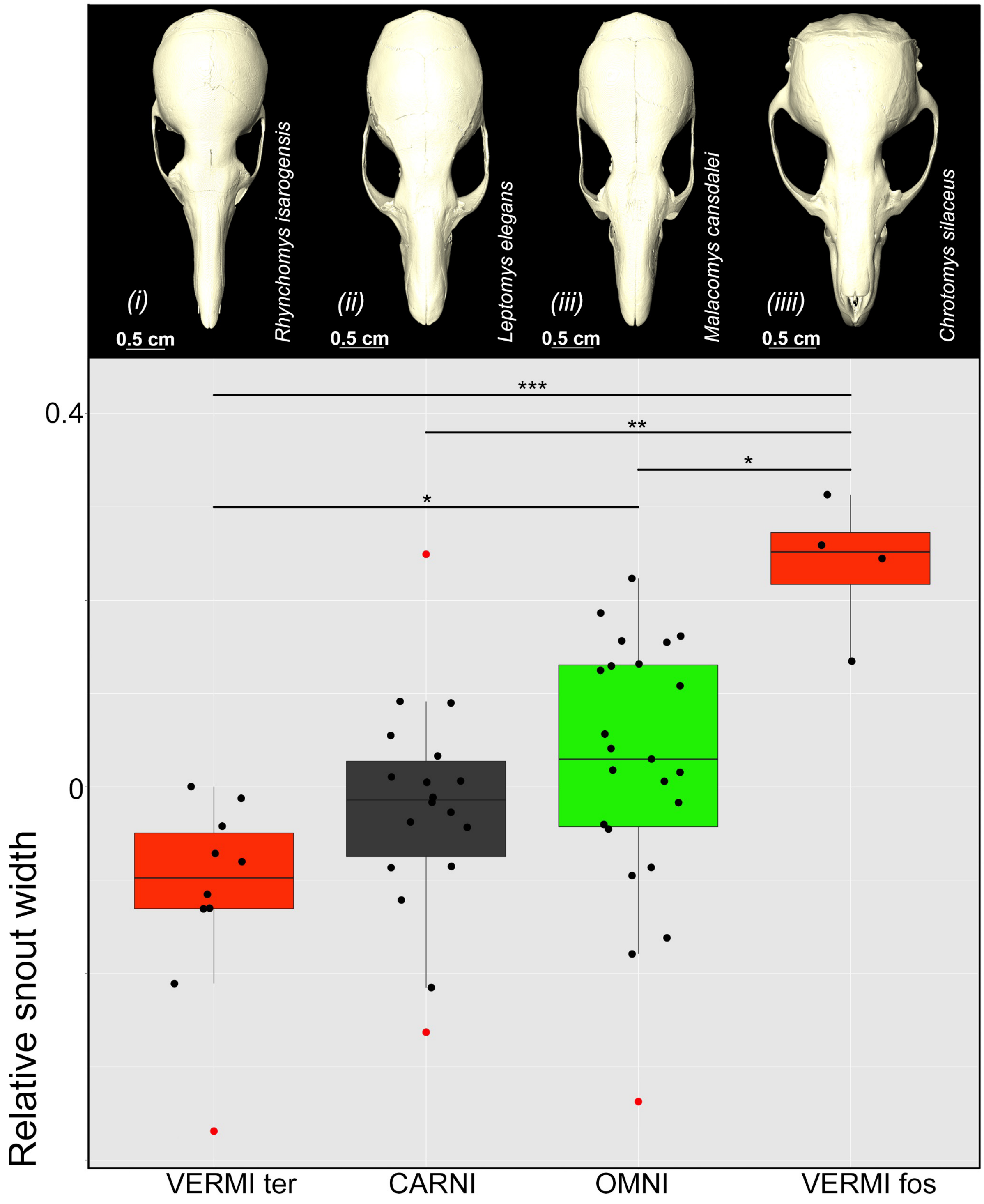

Legend S1. In order to implement phylogenetic comparative analyses to our turbinal dataset, we build a composite phylogenetic tree. Concerning the murinae we used the phylogenetic tree from Rowe et al. (2016). We subsequently used the dated tree from Steppan & Schenk (2017) for the divergence among Cricetidae and the basal split between Muridae and Cricetidae. A species of *Crunomys*, a *Malacomys*, 2 species of *Pseudohydromys*, 3 species of *Leptomys* were missing from these phylogenies. We constrained missing species to their assigned genus, thereby assuming monophyly at the genus level, respectively. We subsequently used the PASTIS R package (Thomas *et al.* 2013) to randomly incorporate them in our composite tree following Maestri *et al.* (2016) protocol.

Table S1. PGLS and linear regression results. Abbreviations: SA = surface area, TotSA = total surface area of turbinals, Relat = relative, Cor = corrected, NoCor = non-corrected, CHAR = convex hull area ratio, CHNSI = convex hull normalized shape index, Respi = respiratory, Olfa = olfactory, NT = nasoturbinal, MT = maxilloturbinal, LS = lamina semicircularis, FT1 = frontoturbinal 1, FT1 bis = frontoturbinal 1 bis, FT2 = frontoturbinal 2, ET1 = ethmoturbinal 1, ET2 = ethmoturbinal 2, and ET3 = ethmoturbinal 3, SKL = skull length, SNW = snout width, SNL = snout length, NaRe = nasoturbinal considered as respiratory turbinal, and NaOl = nasoturbinal considered as olfactory turbinal.

**Variables**	**Slope**	**P-value**	**Signif-code**	**R-squared**	**PGLS slope**	**PGLS p-value**
TotSA/SKL	2.25	2.00e-16	***	0.88	2.30	<0.01
RespiSA/SKL	2.21	2.00e-16	***	0.75	2.26	<0.01
OlfaSA/SKL	2.25	2.00e-16	***	0.88	2.33	<0.01
OlfaSA/RespiSA	0.86	2.00e-16	***	0.83	0.74	<0.01
OlfaSA/TotSA	0.99	2.00e-16	***	0.98	1.00	<0.01
RespiSA/TotSA	1.02	2.00e-16	***	0.92	0.99	<0.01
2DNoCorOlfa/2DNoCorRespi	0.37	2.00e-16	***	0.19	0.33	2.00E-04
2DCorOlfa/SKL	0.34	1.52e-03	**	0.16	0.25	0.08
2DCorRespi/SKL	0.28	0.03	*	0.07	0.21	0.38
2DCorOlfa/RelatOlfaSA	0.41	0.22	-	0.01	0.34	0.16
2DCorRespi/RelatRespiSA	0.48	0.01	**	0.12	0.55	4.00e-03
OlfaCHAR/RespiCHAR	0.29	7.33e-04	***	0.18	0.19	2.80e-03
OlfaCHAR/SKL	0.27	2.60e-05	***	0.27	0.35	1.00e-04
RespiCHAR/SKL	0.19	0.06	-	0.05	0.24	0.18
OlfaCHAR/OlfaSA	0.15	8.21e-10	***	0.50	0.18	<0.01
OlfaCHAR/RelatOlfaSA	0.47	0.02	*	0.08	0.14	0.36
RespiCHAR/RespiSA	0.18	5.33e-07	***	0.36	0.31	<0.01
RespiCHAR/RelatRespiSA	0.55	4.81e-06	***	0.31	0.81	<0.01
OlfaCHNSI/RespiCHNSI	0.40	6.99e-06	***	0.30	0.19	0.01
OlfaCHNSI/SKL	0.17	3.13e-09	***	0.47	0.19	<0.01
RespiCHNSI/SKL	0.15	7.42e-04	***	0.18	0.16	0.06
OlfaCHNSI/OlfaSA	0.08	5.91e-16	***	5.90e-16	0.10	<0.01
OlfaCHNSI/RelatOlfaSA	0.18	0.06	-	0.05	0.10	0.15
RespiCHNSI/RespiSA	0.09	2.55e-09	***	0.48	0.14	<0.01
RespiCHNSI/RelatRespiSA	0.19	4.78e-04	***	0.19	0.35	<0.01

Table S2. Analysis of variance (ANOVA) results for dietary variables. Abbreviations: see Table S1.

Variable	Sum Sq	Mean Sq	F value	Pr(>F)	Signif-code
OlfaSA/TotSA	0.11	0.06	13.04	2.57e-05	***
RespiSA/TotSA	0.67	0.34	15.90	4.11e-06	***
OlfaSA/RespiSA	1.42	0.71	20.57	2.62e-07	***
MT/TotSA	0.73	0.37	7.61	1.25e-03	**
NT/TotSA	0.63	0.31	11.66	6.57e-05	***
2DCorOlfa/SKL	0.66	0.33	15.54	5.11e-06	***
2DCorRespi/SKL	0.18	0.09	1.86	0.17	-
2DNoCorOlfa/2NoDCorRespi	0.03	0.01	17.50	1.54e-06	***
SNL/SKL	0.01	0.01	1.31	0.28	-
SNL/SKL_VERMI_fos_ter	0.04	0.01	3.83	0.01	*
SNW/SNL	0.02	0.01	0.57	0.57	-
SNW/SNL_VERMI_fos_ter	0.38	0.13	8.52	1.09e-04	***
OlfaCHAR/RespiCHAR	0.29	0.15	20.01	3.59e-07	***
OlfaCHAR/SKL	0.18	0.09	11.28	8.54e-05	***
RespiCHAR/SKL	0.12	0.06	2.04	0.14	-
OlfaCHNSI/RespiCHNSI	0.04	0.02	13.66	1.71e-05	***
OlfaCHNSI/SKL	0.02	0.01	6.13	4.08e-03	**
RespiCHNSI/SKL	0.01	0.01	1.32	0.27	-

Table S3. Phylogenetic Tukey's HSD results. Abbreviations: see Table S1.

Variables	Comparison	p-value	Signif-code
RespiSA/TotSA	OMNI/CARNI	0.99	-
RespiSA/TotSA	VERMI/CARNI	1.18e-05	***
RespiSA/TotSA	VERMI/OMNI	1.00e-05	***
OlfaSA/TotSA	OMNI/CARNI	0.97	-
OlfaSA/TotSA	VERMI/CARNI	9.17e-05	***
OlfaSA/TotSA	VERMI/OMNI	8.40e-05	***
OlfaSA/RespiSA	OMNI/CARNI	0.64	-
OlfaSA/RespiSA	VERMI/CARNI	1.00e-04	***
OlfaSA/RespiSA	VERMI/OMNI	1.00e-04	***
MTSA/TotSA	OMNI/CARNI	0.98	-
MTSA/TotSA	VERMI/CARNI	4.47e-03	**
MTSA/TotSA	VERMI/OMNI	1.34e-03	**
NTSA/TotSA	OMNI/CARNI	0.98	-
NTSA/TotSA	VERMI/CARNI	2.67e-04	***
NTSA/TotSA	VERMI/OMNI	2.19e-04	***
SNL/SKL	OMNI/CARNI	0.32	-
SNL/SKL	VERMIter/CARNI	0.94	-
SNL/SKL	VERMIfos/CARNI	0.04	*
SNL/SKL	VERMIter/OMNI	0.19	-
SNL/SKL	VERMIfos/OMNI	0.30	-
SNL/SKL	VERMIter/VERMIfos	0.03	*
SNW/SNL	OMNI/CARNI	0.65	-
SNW/SNL	VERMIter/CARNI	0.21	-
SNW/SNL	VERMIfos/CARNI	2.00e-03	**

Variables	Comparison	p-value	Signif-code
SNW/SNL	VERMIter/OMNI	0.02	*
SNW/SNL	VERMIfos/OMNI	0.01	*
SNW/SNL	VERMIter/VERMIfos	6.03e-05	***
2DCorOlfa/SKL	OMNI/CARNI	2.67e-04	***
2DCorOlfa/SKL	VERMI/CARNI	0.31	-
2DCorOlfa/SKL	VERMI/OMNI	1.00e-04	***
2DCorRespi/SKL	OMNI/CARNI	0.07	-
2DCorRespi/SKL	VERMI/CARNI	0.29	-
2DCorRespi/SKL	VERMI/OMNI	0.88	-
2DNoCorOlfa/2NoDCorRespi	OMNI/CARNI	0.52	-
2DNoCorOlfa/2NoDCorRespi	VERMI/CARNI	1.00e-04	***
2DNoCorOlfa/2NoDCorRespi	VERMI/OMNI	1.00e-04	***
OlfaCHAR/RespiCHAR	OMNI/CARNI	0.99	-
OlfaCHAR/RespiCHAR	VERMI/CARNI	2.70e-06	***
OlfaCHAR/RespiCHAR	VERMI/OMNI	1.60e-06	***
OlfaCHAR/SKL	OMNI/CARNI	0.05	*
OlfaCHAR/SKL	VERMI/CARNI	0.06	-
OlfaCHAR/SKL	VERMI/OMNI	5.42e-05	***
OlfaCHNSI/RespiCHNSI	OMNI/CARNI	0.25	-
OlfaCHNSI/RespiCHNSI	VERMI/CARNI	1.36e-05	***
OlfaCHNSI/RespiCHNSI	VERMI/OMNI	8.26e-04	***
OlfaCHNSI/SKL	OMNI/CARNI	0.39	
OlfaCHNSI/SKL	VERMI/CARNI	0.09	-
OlfaCHNSI/SKL	VERMI/OMNI	2.73e-03	**

Table S4. Phylogenetic Tukey's HSD differences between considering the nasoturbinal whether as olfactory whether as respiratory turbinals. Abbreviations: see Table S1.

Variables	Comparison	NaRe p-value	NaOl p-value
RespiSA/TotSA	OMNI/CARNI	0.76	0.83
RespiSA/TotSA	VERMI/CARNI	0.01	0.03
RespiSA/TotSA	VERMI/OMNI	0.00	0.00
OlfaSA/TotSA	OMNI/CARNI	0.90	1.00
OlfaSA/TotSA	VERMI/CARNI	0.01	0.03
OlfaSA/TotSA	VERMI/OMNI	0.00	0.04
OlfaSA/RespiSA	OMNI/CARNI	0.86	0.75
OlfaSA/RespiSA	VERMI/CARNI	0.00	0.00
OlfaSA/RespiSA	VERMI/OMNI	0.00	0.00

Table S5. Comparison between the results of phylogenetic Tukey's HSD and non-phylogenetic Tukey's HSD. Abbreviations: see Table S1.

Variables	Comparison	PhyloTukey p-value	Tukey p-value
RespiSA/TotSA	OMNI/CARNI	0.76	0.94
RespiSA/TotSA	VERMI/CARNI	0.01	0.00
RespiSA/TotSA	VERMI/OMNI	0.00	0.00
OlfaSA/TotSA	OMNI/CARNI	0.90	0.98
OlfaSA/TotSA	VERMI/CARNI	0.01	0.00
OlfaSA/TotSA	VERMI/OMNI	0.00	0.00
OlfaSA/RespiSA	OMNI/CARNI	0.86	0.44
OlfaSA/RespiSA	VERMI/CARNI	0.00	0.00
OlfaSA/RespiSA	VERMI/OMNI	0.00	0.00

Table S6. Results of phylogenetic ANCOVA. Three models were contrasted: a model without dietary categories (H0), a model with omnivorous and carnivorous dietary categories (Carni), and a model with omnivorous, carnivorous, and vermivorous dietary categories (Vermi). Model were compared using the Akaike information criterion (AIC) and the Likelihood-ratio test.

	**AIC**	**Lr test likelihood**	**Lr test p-value**		
	RespiSA/TotSA		H0	Carni	Vermi
H0	512.34	-253.17	-	2.12e-04	6.02e-09
Carni	499.42	-244.71	-	-	8.46e-07
Vermi	477.17	-232.58	-	-	-

	**AIC**	**Lr test likelihood**	**Lr test p-value**		
	OlfaSA/TotSA		H0	Carni	Vermi
H0	512.34	-253.17	-	2.12e-04	6.02e-09
Carni	499.42	-244.71	-	-	8.46e-07
Vermi	477.17	-232.58	-	-	-

	**AIC**	**Lr test likelihood**	**Lr test p-value**		
	OlfaSA/RespiSA		H0	Carni	Vermi
H0	636.07	-315.03	-	3.20e-06	2.06e-12
Carni	614.76	-302.38	-	-	1.43e-08
Vermi	584.62	-286.31	-	-	-

	**AIC**	**Lr test likelihood**	**Lr test p-value**		
	RespiSKL		H0	Carni	Vermi
H0	565.52	-279.76	-	1.55e-05	5.06e-09
Carni	591.67	-290.84	-	-	1.47e-15
Vermi	530.00	-259.00	-	-	-

	**AIC**	**Lr test likelihood**	**Lr test p-value**		
	OlfaSKL		H0	Carni	Vermi
H0	636.07	-315.03	-	3.10e-11	2.48e-12
Carni	591.67	-290.84	-	-	3.22e-03
Vermi	585.00	-286.50	-	-	-

	**AIC**	**Lr test likelihood**	**Lr test p-value**		
	OlfaCHAR/RespiCHAR		H0	Carni	Vermi
H0	59.32	-26.66	-	9.84e-13	4.98e-14
Carni	8.03	0.99	-	-	1.82e-03
Vermi	0.31	5.85	-	-	-

Table S7. Results of 1.000 simulations of single-rate BM and three alternative OU models with the ratio between snout width (SNW) and snout length (SNL). BM and OU1 with omnivorous and all carnivorous dietary categories (carnivorous + vermivorous); OU2 with omnivorous, carnivorous, and vermivorous dietary categories; and OU3 with omnivorous, carnivorous, terrestrial vermivorous, and semi-fossorial vermivorous dietary categories. AICc = Akaike's information criterion corrected. ∆AICc = difference between AICc compared to minimum AICc.

	**SNW/SNL**	
Model	AICc	∆AICc
**BM**	-90.22	27.60
**OU1**	-104.65	13.17
**OU2**	-104.60	13.21
**OU3**	-117.82	00.00

Table S8. Dataset used for quantitative analyses. Abbreviations: see Table S1. American Museum of Natural History (AMNH), Centre de Biologie et de Gestion des Populations (CBGP), Field Museum of Natural History (FMNH), Museums Victoria (NMV), Museum Zoologicum Bogoriense (MZB), Natural History Museum London (NHMUK previously BMNH), Natural History Museum of Paris (MNHN), Smithsonian Institution National Museum of Natural History (NMNH), and University of Montpellier (UM).

Taxa	MUSEUM	VOUCHER	Taxa_2	DIET	Sexe	mtSA	ntSA	lsSA	ft1SA	etISA	ft2SA	etIISA	etIIISA	TotSA	SKL	SNL	SNW	2DRespi	2DOlfa	2DCorRespi	2DCorOlfa	ChARRespi	ChAROlfa	ChNSIRespi	ChNSIOlfa
Apomys_abrae	AMNH	242099,00	APOabra	OMNI	-	45,86	112,45	25,73	54,49	73,22	36,87	50,99	54,84	454,45	37,00	13,40	6,09	1,47	1,52	2498,64	3683,00	1,47	2,14	1,54	1,72
Apomys_banahao	CBGP	P0045	APObana	OMNI	-	71,18	93,17	22,40	55,86	84,52	40,42	50,63	62,37	480,56	36,51	13,08	6,50	1,53	1,53	2768,21	4242,00	1,48	2,29	1,59	1,76
Apomys_banahao	CBGP	P0067	APObana	OMNI	-	51,74	82,52	21,15	41,47	66,34	32,26	35,17	43,19	373,83	33,74	12,16	5,65	-	-	-	-	1,48	2,03	1,55	1,63
Apomys_banahao	CBGP	P0066	APObana	OMNI	-	95,83	141,24	27,78	60,51	89,26	39,57	43,07	71,20	568,47	38,38	13,30	6,81	-	-	-	-	1,67	2,17	1,66	1,70
Apomys_datae	FMNH	252475,00	APOdata	OMNI	F	57,64	143,09	42,91	80,95	126,60	60,02	69,49	88,33	669,03	38,48	14,26	7,62	1,56	1,56	3277,09	5127,00	1,62	2,74	1,65	1,89
Apomys_datae	FMNH	188256,00	APOdata	OMNI	M	72,04	110,79	31,89	82,47	117,05	55,07	59,41	80,02	608,73	37,73	13,95	6,54	-	-	-	-	1,32	2,48	1,49	1,83
Apomys_hylocetes	USNM	125244,00	APOhylo	OMNI	F	39,44	93,68	13,59	32,53	54,47	18,96	28,01	36,11	316,78	30,51	11,02	5,40	1,48	1,46	2500,00	3709,00	1,95	2,22	1,84	1,71
Apomys_littoralis	USNM	458755,00	APOlitt	OMNI	M	35,44	60,65	13,42	32,47	58,77	20,12	29,07	35,99	285,94	29,14	9,34	5,21	1,46	1,42	2191,68	3197,00	1,47	2,10	1,58	1,69
Archboldomys_luzonensis	USNM	573840,00	ARCluzo	INSEC	F	33,27	48,03	15,44	38,81	52,07	19,01	26,29	33,29	266,21	26,67	9,10	5,13	1,46	1,53	2695,39	3935,00	1,51	2,45	1,58	1,79
Archboldomys_luzonensis	USNM	573837,00	ARCluzo	INSEC	M	32,78	52,09	12,07	38,71	54,39	19,37	27,30	37,88	274,58	28,10	9,90	4,93	-	-	-	-	1,42	2,42	1,56	1,79
Bunomys_andrewsi	NMV	36977,00	BUNandr	OMNI	-	62,54	132,80	21,63	59,70	80,96	45,31	50,49	66,65	520,08	36,68	15,55	6,63	1,43	1,46	2083,80	2979,00	1,68	2,32	1,72	1,77
Bunomys_andrewsi	NMV	36982,00	BUNandr	OMNI	-	88,70	150,71	34,02	64,89	100,92	43,97	53,13	61,15	597,48	40,75	16,13	7,04	-	-	-	-	1,70	2,18	1,74	1,72
Bunomys_penitus	NMV	36984,00	BUNpeni	OMNI	-	77,80	179,94	42,61	105,51	137,21	86,21	115,81	109,92	855,00	45,23	18,67	8,31	1,53	1,58	2296,93	3512,00	1,55	2,64	1,67	1,86
Chrotomys_gonzalesi	USNM	458955,00	CHRgonz	WORM	F	50,11	53,48	24,36	80,38	78,84	37,79	51,63	60,37	436,95	34,45	12,00	7,49	1,36	1,52	2238,40	3044,00	1,16	2,43	1,43	1,80
Chrotomys_mindorensis	FMNH	222107,00	CHRmind	WORM	F	43,34	85,29	38,74	94,64	91,61	49,60	70,22	86,33	559,77	37,54	12,52	8,19	1,36	1,55	2010,33	2725,00	1,32	2,74	1,50	1,88
Chrotomys_silaceus	NHMUK	97.5.2.16	CHRsila	WORM	M	47,19	61,08	24,81	68,76	75,60	38,55	51,14	49,98	417,10	34,62	11,06	6,91	1,48	1,53	2644,03	3924,00	1,50	2,33	1,58	1,71
Chrotomys_silaceus	NHMUK	97.5.2.39	CHRsila	WORM	F	41,72	59,29	18,89	56,20	61,89	37,46	51,64	56,79	383,87	33,31	11,21	6,02	-	-	-	-	1,53	2,40	1,63	1,77
Chrotomys_whiteadi	NHMUK	95.8.2.20	CHRwhit	WORM	F	49,03	76,45	28,03	82,65	105,97	51,56	62,95	90,61	547,26	35,29	12,30	7,68	1,42	1,60	2683,92	3805,00	1,57	2,88	1,62	1,95
Chrotomys_whiteadi	NHMUK	95.8.2.19	CHRwhit	WORM	M	64,47	85,89	38,71	94,05	92,76	56,15	67,34	91,50	590,87	36,84	12,12	7,59	-	-	-	-	1,44	2,53	1,55	1,85
Chrotomys_whiteadi	NHMUK	97.5.2.17	CHRwhit	WORM	F	57,88	80,57	33,00	80,92	83,95	47,17	53,42	79,17	516,08	35,78	12,24	7,73	-	-	-	-	1,38	2,48	1,56	1,82
Crunomys_fallax	NHMUK	97.4.8.4	CRUfall	INSEC	-	15,78	22,72	9,17	16,24	24,17	9,69	12,78	16,09	126,63	25,89	8,82	4,06	1,28	1,43	1256,45	1606,00	0,94	1,85	1,35	1,56
Crunomys_melanius	NHMUK	7.2.2.14	CRUmela	INSEC	M	23,39	29,81	8,93	23,20	35,78	13,63	19,14	24,01	177,89	27,12	9,24	6,61	1,37	1,39	1841,28	2515,00	1,06	1,94	1,38	1,57
Deomys_ferrugineus	MNHN	1620,00	DEOferr	INSEC	-	46,18	100,29	13,22	24,11	79,92	29,51	39,54	41,62	374,38	37,05	16,04	5,22	1,45	1,56	2271,12	3282,00	1,62	2,23	1,62	1,73
Deomys_ferrugineus	MNHN	1622,00	DEOferr	INSEC	-	49,53	81,63	14,48	23,50	63,38	22,92	36,12	34,94	326,49	35,84	15,29	5,07	-	-	-	-	1,60	2,15	1,61	1,70
Echiothrix_centrosa	NHMUK	40.385	ECHcent	WORM	F	95,13	152,01	39,44	106,40	142,58	62,49	81,37	112,51	791,92	51,82	21,44	7,19	1,45	1,55	2520,53	3653,00	1,54	2,32	1,71	1,83
Echiothrix_centrosa	AMNH	153013,00	ECHcent	WORM	M	91,34	132,22	42,59	93,33	143,38	56,80	70,30	94,85	724,82	52,29	22,83	6,75	-	-	-	-	1,40	2,41	1,63	1,83
Echiothrix_leucura	NHMUK	97.1.2.46	ECHleuc	WORM	M	96,81	159,22	46,65	101,20	152,26	55,06	82,46	90,03	783,69	53,68	22,50	6,95	1,43	1,56	2420,25	3452,00	1,39	2,36	1,68	1,82
Echiothrix_leucura	NHMUK	97.1.2.45	ECHleuc	WORM	M	93,01	128,75	41,30	107,86	126,19	68,16	86,23	119,37	770,86	53,13	22,54	7,12	-	-	-	-	1,40	2,38	1,66	1,80
Gracillimus_radix	NMV	36995,00	GRAradi	OMNI	-	24,73	41,67	12,16	29,49	54,12	17,93	24,24	20,35	224,69	29,51	10,00	5,12	1,38	1,47	1876,77	2583,00	1,25	2,22	1,43	1,68
Halmaheramys_bokimekot	MZB	33266,00	HALboki	OMNI	-	116,12	178,48	50,38	103,91	149,48	68,35	85,72	82,56	834,99	47,52	18,26	8,43	1,49	1,53	2475,95	3681,00	1,72	2,45	1,71	1,83
Hyorhinomys_stuempkei	NMV	C37198	HYOstue	WORM	-	45,62	73,22	24,02	82,33	98,39	58,87	73,39	91,13	546,97	46,59	18,35	7,15	1,29	1,49	1898,19	2450,00	1,21	2,57	1,45	1,80
Leptomys_arfakensis	NHMUK	29.5.27.22	LEParf	INSEC	F	47,48	86,64	21,57	53,28	81,39	38,61	46,87	50,30	426,14	38,98	14,31	6,43	1,44	1,56	2234,15	3225,00	1,53	2,34	1,53	1,75
Leptomys_elegans	NHMUK	5.11.28.22	LEPeleg	INSEC	M	51,69	91,06	26,38	49,94	71,44	35,71	41,03	54,68	421,91	36,79	13,93	6,32	1,50	1,52	2490,17	3738,00	1,55	2,19	1,62	1,76
Leptomys_elegans	NHMUK	50.1254	LEPeleg	INSEC	M	51,95	75,32	27,63	47,17	66,37	34,68	41,22	55,87	400,20	36,01	12,74	6,17	-	-	-	-	1,50	2,22	1,56	1,74
Leptomys_ernstmayri	NHMUK	50.1252	LEPerns	INSEC	M	60,87	92,89	26,08	56,28	76,00	42,67	44,16	49,29	448,22	36,67	13,70	6,30	1,48	1,54	2848,95	4221,00	1,64	2,46	1,60	1,79
Leptomys_ernstmayri	AMNH	194936,00	LEPerns	INSEC	F	46,79	71,36	24,83	41,69	59,80	29,08	35,72	44,07	353,33	35,55	11,91	6,22	-	-	-	-	1,40	2,26	1,51	1,73
Leptomys_signatus	AMNH	105370,00	LEPsign	INSEC	F	44,43	64,80	17,93	32,67	56,63	26,42	30,50	38,62	312,00	33,40	12,02	5,62	1,46	1,47	2384,04	3490,00	1,42	2,06	1,54	1,66
Malacomys_cansdalei	USNM	486151,00	MALcans	OMNI	M	57,40	91,74	29,17	78,86	84,95	42,11	62,43	74,79	521,46	38,98	15,14	6,70	1,41	1,58	2271,92	3210,00	1,36	2,60	1,62	1,88
Malacomys_edwardsi	USNM	467226,00	MALedwa	OMNI	F	37,65	80,22	15,26	50,81	61,99	21,35	31,80	44,34	343,43	33,69	13,41	5,18	1,45	1,54	2206,53	3203,00	1,28	2,27	1,53	1,78
Maxomys_musschenbroekii	NMV	37009,00	MAXmuss	OMNI	-	89,41	121,07	26,45	77,42	102,55	41,92	62,22	96,41	617,44	42,53	16,11	6,17	1,48	1,55	2262,00	3355,00	1,46	2,40	1,56	1,82
Maxomys_surifer	CBGP	6369,00	MAXsuri	OMNI	F	117,11	91,74	35,17	87,11	109,53	52,84	67,63	90,47	651,60	44,98	16,59	7,50	1,46	1,49	2493,32	3641,00	1,38	2,14	1,61	1,73
Maxomys_surifer	CBGP	C0119	MAXsuri	OMNI	F	115,37	119,17	39,25	108,19	137,39	53,23	81,92	98,94	753,47	44,29	17,64	7,30	1,33	1,47	1986,61	2640,00	1,17	2,52	1,50	1,86
Mayermys_ellermani	AMNH	53278,00	MAYelle	INSEC	M	11,77	18,40	6,14	14,23	19,11	8,45	11,68	10,37	100,15	21,40	6,84	4,54	1,33	1,47	1986,61		1,39	2,00	1,48	1,59
Mayermys_ellermni	NHMUK	53280,00	MAYelle	INSEC	M	16,30	22,38	6,82	19,28	25,57	13,02	14,40	14,55	132,32	22,79	6,58	5,08	-	-	-	-	1,32	2,16	1,46	1,65
Melasmothrix_naso	AMNH	225092,00	MELnaso	WORM	F	32,23	45,41	14,56	39,27	47,34	24,26	28,20	37,01	268,29	31,87	11,49	5,29	1,44	1,55	2426,67	3483,00	1,29	2,28	1,57	1,70
Melasmothrix_naso	AMNH	225101,00	MELnaso	WORM	M	30,17	50,35	18,44	48,13	48,91	29,94	34,06	43,01	303,01	32,50	12,59	5,18	-	-	-	-	1,34	2,45	1,60	1,76
Melomys_burtoni	MZBM	33533,00	MELburt	OMNI	M	53,59	99,89	18,23	50,88	58,27	24,89	34,72	53,77	394,25	34,23	10,97	7,14	1,47	1,47	2285,68	3354,00	1,62	2,07	1,39	1,69
Melomys_burtoni	MZBM	33552,00	MELburt	OMNI	M	41,35	67,88	18,06	46,42	51,18	19,97	27,58	48,18	320,62	32,53	10,34	5,79	-	-	-	-	1,40	2,09	1,51	1,72
Melomys_obiensis	MZB	YS394	MELobie	OMNI	-	37,06	50,77	15,71	31,96	32,59	18,73	22,56	36,30	245,66	33,60	10,19	4,60	1,38	1,44	1957,81	2710,00	1,22	1,79	1,51	1,59
Microhydromys_richardsoni	AMNH	198790,00	MICrich	INSEC	M	10,74	13,92	6,31	13,65	17,89	9,90	11,63	14,01	98,04	20,00	6,26	4,20	1,33	1,47	1703,44	2270,00	1,15	1,93	1,41	1,58
Mus_cervicolor	CBGP	7314,00	MUScerv	OMNI	M	11,91	15,54	5,47	14,79	18,78	7,54	8,85	18,89	101,77	21,54	7,25	4,19	1,32	1,41	1492,22	1966,00	0,97	1,81	1,34	1,55
Mus_pahari	CBGP	7226,00	MUSpaha	INSEC	-	25,81	35,12	18,50	39,10	37,61	18,78	22,19	36,29	233,40	25,78	9,54	4,80	1,40	1,55	1753,60	2461,00	1,22	2,36	1,56	1,75
Nannomys_minutoides	UM	–	NANminu	OMNI	F	6,55	11,37	3,90	8,14	11,92	3,91	7,48	13,27	66,55	18,98	6,69	3,09	1,28	1,35	1384,58	1776,00	0,98	1,75	1,33	1,54
Oxymycterus_dasytrichus	MNHN	CG1468N2002	OXYdasy	INSEC	-	37,93	93,26	25,30	50,03	88,44	40,81	53,83	66,45	456,06	37,89	15,18	6,05	1,48	1,57	2882,11	4271,00	1,52	2,65	1,68	1,86
Oxymycterus_quaestor	MNHN	272,00	OXYquae	INSEC	-	55,83	124,73	22,21	46,87	88,37	39,28	50,28	50,04	477,62	36,44	13,29	6,76	1,40	1,57	2145,41	3004,00	1,80	2,49	1,70	1,79
Paraleptomys_wilhelmina	AMNH	150546,00	PARwilh	INSEC	F	57,60	74,97	17,80	42,13	55,68	26,06	33,33	41,38	348,95	31,62	11,87	5,55	1,48	1,50	3054,22	4512,00	1,90	2,33	1,71	1,74
Paraleptomys_wilhelmina	AMNH	150536,00	PARwilh	INSEC	M	51,18	66,10	11,64	38,81	49,72	26,21	31,53	38,95	314,13	31,47	10,82	5,67	-	-	-	-	1,61	2,13	1,61	1,67
Paucidentomys_vermidax	FMNH	213102,00	PAUverm	WORM	-	30,11	61,40	23,37	58,34	102,36	57,30	64,13	90,87	487,88	53,37	21,50	5,73	1,33	1,59	2013,04	2687,00	0,94	2,50	1,37	1,82
Paucidentomys_vermidax	MZB	35001,00	PAUverm	WORM	-	21,62	45,13	19,46	53,52	79,97	44,82	54,04	69,93	388,50	42,26	17,30	4,96	-	-	-	-	1,16	2,60	1,56	1,88
Paucidentomys_vermidax	NMV	HH766	PAUverm	WORM	-	24,35	50,44	19,47	47,67	72,53	48,15	55,21	77,46	395,29	44,16	17,93	4,55	-	-	-	-	1,07	2,56	1,49	1,86
Pseudohydromys_eleanorae	NHMUK	50.1734	PSEelea	INSEC	F	18,39	25,56	7,33	12,66	21,41	10,48	12,89	11,36	120,08	22,39	7,42	4,65	1,37	1,42	2059,68	2823,00	1,32	1,93	1,47	1,55
Pseudohydromys_fuscus	NHMUK	53.296	PSEfusc	INSEC	M	19,31	28,79	9,20	25,30	33,70	10,50	19,30	23,00	169,10	23,92	8,30	5,13	1,44	1,46	2286,55	3302,00	1,32	2,18	1,50	1,67
Pseudohydromys_fuscus	NHMUK	501.733	PSEfusc	INSEC	M	18,44	29,80	8,04	16,72	23,98	10,84	15,01	15,50	138,32	23,06	7,65	4,19	-	-	-	-	1,43	2,00	1,53	1,61
Pseudohydromys_murinus	NHMUK	53.290	PSEmuri	INSEC	F	11,88	23,17	9,30	19,12	30,93	12,61	17,54	15,68	140,23	22,57	6,90	4,71	1,35	1,45	1763,01	2382,00	1,30	2,05	1,42	1,62
Rattus_marmosurus	NMV	37018,00	RATmarm	OMNI	-	52,67	57,23	31,67	48,28	88,67	33,32	51,49	58,98	422,30	45,38	15,75	8,39	-	-	-	-	0,77	1,89	1,22	1,62
Rattus_morotaiensis	MZB	33229,00	RATmoro	OMNI	F	48,40	78,74	21,88	44,03	71,04	29,49	34,87	54,81	383,27	37,16	12,39	6,75	1,41	1,48	1664,89	2343,00	1,19	1,80	1,53	1,65
Rattus_norvegicus	CBGP	C0304	RATnorv	OMNI	M	38,80	47,79	16,51	30,78	51,76	31,74	34,42	46,00	297,79	37,95	12,70	6,88	1,34	1,49	1690,10	2272,00	0,99	2,01	1,40	1,67
Rattus_praetor	CBGP	-	RATprae	OMNI	-	55,63	92,05	23,12	47,66	95,30	27,36	32,94	26,46	400,52	41,02	14,20	7,68	1,41	1,49	2080,65	2941,00	1,35	1,98	1,55	1,65
Rattus_tanezumi	CBGP	R5422	RATtane	OMNI	F	42,37	70,92	16,96	40,14	75,38	26,89	38,30	50,74	361,69	37,09	13,06	6,30	1,39	1,50	2081,78	2897,00	1,46	2,21	1,60	1,78
Rhynchomys_isarogensis	USNM	573576,00	RHYisar	WORM	M	31,54	80,86	17,16	46,29	80,29	41,99	54,23	70,10	422,47	39,24	15,77	6,50	1,44	1,59	2767,72	3975,00	1,56	2,68	1,69	1,84
Rhynchomys_isarogensis	USNM	573585,00	RHYisar	WORM	M	40,66	98,02	24,71	76,79	119,08	71,19	78,65	102,74	611,84	43,85	17,16	7,18	-	-	-	-	1,44	3,02	1,66	1,96
Rhynchomys_isarogensis	FMNH	573913,00	RHYisar	WORM	M	36,87	98,84	27,58	64,62	106,67	58,57	63,64	97,24	554,02	42,93	16,83	6,98	-	-	-	-	1,50	2,75	1,67	1,87
Rhynchomys_soricoides	NHMUK	95.8.2.18	RHYsori	WORM	M	35,07	91,22	21,90	60,24	95,80	53,69	67,30	75,87	501,09	45,25	17,69	6,82	1,36	1,58	1887,43	2572,00	1,31	2,63	1,63	1,86
Rhynchomys_soricoides	NHMUK	97.5.2.18	RHYsori	WORM	M	38,10	128,98	37,71	100,52	161,78	73,10	91,00	119,72	750,92	48,69	19,46	7,31	-	-	-	-	1,43	3,08	1,74	2,02
Rhynchomys_soricoides	FMNH	193833,00	RHYsori	WORM	F	31,63	97,66	24,63	83,05	110,40	60,88	67,45	107,07	582,76	48,58	19,72	6,25	-	-	-	-	1,34	2,72	1,67	1,92
Rhynchomys_soricoides	NHMUK	95.8.2.17	RHYsori	WORM	M	38,30	114,72	29,58	99,90	143,69	71,95	86,70	127,29	712,12	48,24	19,55	7,12	-	-	-	-	1,41	2,92	1,68	1,97
Sigmodon_hispidus	MNHN	CG2007N335	SIGhisp	OMNI	-	38,14	45,59	12,51	32,67	75,33	21,20	30,43	40,74	296,61	36,67	13,40	7,28	1,34	1,43	1477,09	1979,00	1,11	2,02	1,37	1,66
Sommeromys_macrorhinus	NMV	KCR2644	SOMmacr	INSEC	-	91,09	93,97	16,48	48,25	53,70	22,76	27,44	45,22	398,91	33,37	13,03	5,27	1,51	1,54	2936,30	4425,00	2,03	2,28	1,84	1,72
Sommeromys_macrorhinus	NMV	37074,00	SOMmacr	INSEC	-	75,86	79,84	16,41	36,97	38,70	18,95	20,12	35,56	322,41	31,94	12,30	5,23	-	-	-	-	1,85	2,10	1,75	1,63
Sommeromys_macrorhinus	AMNH	M226956	SOMmacr	INSEC	M	77,99	109,36	19,34	53,91	72,19	31,13	39,05	56,11	459,08	35,07	13,35	5,72	-	-	-	-	1,85	2,39	1,77	1,76
Sommeromys_macrorhinus	NMV	KCR2596	SOMmacr	INSEC	-	105,82	92,89	16,25	50,93	54,00	22,34	27,04	45,58	414,85	33,29	11,84	5,09	-	-	-	-	2,04	2,22	1,84	1,70
Soricomys_leonardocoi	FMNH	190966,00	SORleon	WORM	F	15,59	25,13	8,74	24,40	31,06	12,57	17,87	21,89	157,25	24,60	7,83	4,55	1,37	1,54	1446,52	1980,00	1,15	2,48	1,37	1,77
Tateomys_macrocercus	AMNH	225073,00	TATmacr	WORM	F	43,04	61,88	20,19	39,24	51,62	24,43	30,55	43,83	314,76	30,65	10,73	5,28	1,42	1,57	2304,43	3261,00	1,66	2,47	1,67	1,77
Tateomys_macrocercus	AMNH	225072,00	TATmacr	WORM	F	44,58	58,75	18,66	42,76	51,51	26,38	31,43	42,85	316,92	32,29	11,42	5,34	-	-	-	-	1,46	2,28	1,60	1,71
Tateomys_macrocercus	NMV	37081,00	TATmacr	WORM	-	37,62	52,46	16,56	40,72	53,10	24,52	30,87	45,85	301,69	32,43	12,13	5,23	-	-	-	-	1,55	2,41	1,58	1,76
Tateomys_rhinogradoides	NMV	KCR2634	TATrhin	WORM	-	41,81	58,63	24,99	61,80	86,40	46,39	60,70	68,54	449,26	39,85	16,88	5,16	1,38	1,59	1823,98	2518,00	1,34	2,60	1,58	1,84
Tateomys_rhinogradoides	AMNH	M225117	TATrhin	WORM	M	38,90	69,21	31,62	67,80	104,74	38,92	59,16	65,53	475,86	40,09	14,16	5,63	-	-	-	-	1,45	2,91	1,62	1,93

Table S9. List of references for dietary categorizations.

Species	DIET	Region	Reference
APOabra	OMNI	PHIL	Heaney et. al. 2016
APObana	OMNI	PHIL	Heaney et. al. 2016
APOdata	OMNI	PHIL	Heaney et. al. 2016
APOhylo	OMNI	PHIL	L. Heaney pers. comm.; IUCN red list
APOlitt	OMNI	PHIL	Based on other Apomys
ARCluzo	CARNI	PHIL	Rickart et. al. 1991; Heaney et. al. 1999; Balete et. al. 2012
BUNandr	OMNI	SULAW	Musser 2014
BUNpeni	OMNI	SULAW	Musser 2014
CHRgonz	VERMI	PHIL	Rickart et. al. 1991; Heaney et. al. 1999
CHRmind	VERMI	PHIL	Rickart et. al. 1991; Heaney et. al. 1999; Heaney et. al. 2016
CHRsila	VERMI	PHIL	Heaney et. al. 2010; Rickart et. al. 2011
CHRwhit	VERMI	PHIL	Heaney et. al. 2010; Rickart et. al. 2011
CRUfall	CARNI	PHIL	-
DEOferr	CARNI	AFRIC	Happold 2013
CRUmela	CARNI	PHIL	Musser & Durden 2002
ECHcent	VERMI	SULAW	Musser & Durden 2014
ECHleuc	VERMI	SULAW	Musser 1990
GRAradi	OMNI	SULAW	Rowe et. al. 2016
HALboki	OMNI	MOLUC	Fabre et. al. 2013
HYOstue	VERMI	SULAW	Esselstyn et. al. 2015
LEParf	CARNI	PAPUA	Musser et. al. 2008
LEPeleg	CARNI	PAPUA	Musser et. al. 2008
LEPerns	CARNI	PAPUA	Musser et. al. 2008
LEPsign	CARNI	PAPUA	Musser et. al. 2008
MALedwa	OMNI	AFRIC	Happold 1987; Cole 1975
MAXmuss	OMNI	SULAW	Musser 1982
MAXsuri	OMNI	ORIEN	Pimsai et. al. 2014
MELburt	OMNI	MOLUC	Kerle 2008
MELobie	OMNI	MOLUC	Pers. Data.
MELnaso	VERMI	SULAW	Musser 1982
MICrich	CARNI	PAPUA	Helgen et. al. 2010
MUScerv	OMNI	ORIEN	Francis 2008; Lekagul & McNeely 1988
MUSpaha	CARNI	ORIEN	Smith & Xie 2008
NANminu	OMNI	AFRIC	Happold 2013
OXYdasy	CARNI	AMERI	Based on other Oxymycterus species; Wilson et. al. 2017
OXYquae	CARNI	AMERI	Based on other Oxymycterus species; Wilson et. al. 2017
PARwilh	CARNI	PAPUA	Musser et. al. 2008
PAUverm	VERMI	SULAW	Esselstyn et. al. 2012
PSEelea	CARNI	PAPUA	Based on other Pseudohydromys
PSEelle	CARNI	PAPUA	Based on other Pseudohydromys
PSEfusc	CARNI	PAPUA	Wilson et. al. 2017
PSEmuri	CARNI	PAPUA	Wilson et. al. 2017
RATmarm	OMNI	SULAW	Musser 1982
RATmoro	OMNI	MOLUC	Pers. Comm.
RATnorv	OMNI	ORIEN	Francis 2008; Zhang et. al. 2005
RATprae	OMNI	PAPUA	Taylor et. al. 1982; Flannery 1995
RATtane	OMNI	ORIEN	Pimsai et. al. 2014
RHYisar	VERMI	PHIL	Rickart et. Al. 1991; Heaney et. al. 1999; Rickart et. al. 2011; Balete et. al. 2009
RHYsori	VERMI	PHIL	Rickart et. Al. 1991; Heaney et. al. 1999; Rickart et. al. 2011; Balete et. al. 2009
SIGhisp	OMNI	AMERI	Cameron & Spencer 1981
SOMmacr	CARNI	SULAW	Musser & Durden 2002; Achmadi et. al. 2014
SORleon	VERMI	PHIL	Balete et. al. 2012
TATmacr	VERMI	SULAW	Musser 1982
TATrhin	VERMI	SULAW	Musser 1982
